# Tethering Efficiency of Reversible Addition‐Fragmentation Chain Transfer‐Synthesized Styrene Maleic Acid Polymers and Associated Styrene Maleic Acid Lipid Nanoparticles on Gold Surfaces

**DOI:** 10.1002/cplu.202500173

**Published:** 2025-04-17

**Authors:** Michelle D. Farrelly, Denis Korneev, Lisandra L. Martin, San H. Thang

**Affiliations:** ^1^ School of Chemistry Monash University Clayton VIC 3800 Australia; ^2^ Ramaciotti Centre for Cryo‐Electron Microscopy Monash University Clayton VIC 3800 Australia; ^3^ School of BioSciences and Bio21 Molecular Sciences and Biotechnology Institute The University of Melbourne Parkville VIC 3010 Australia

**Keywords:** biological membranes, functionalised copolymers, gold affinity, reversible addition-fragmentation chain transfer, styrene maleic acid lipid nanoparticles, surface‐immobilisation, trithiocarbonate moiety

## Abstract

Styrene maleic acid lipid nanoparticles (SMALPs) arise from amphipathic styrene maleic acid (SMA) copolymer encapsulation of membranes into polymer‐lipid nanodiscs, structures applied in the native extraction of membrane proteins (MPs). Strategies to immobilize SMALPs via their polymer belt onto surfaces allow the biophysical study of MPs without direct protein‐surface anchoring. In this work, reversible addition‐fragmentation chain transfer (RAFT) polymerization is used to synthesize a library of diblock SMA copolymers to determine the optimal sequence for SMALP assembly. The further ability of trithiocarbonates (T) and attached (Z)‐end‐groups, generated by RAFT polymerization, to tether SMALPs to gold surfaces via sulfur‐gold bonds is evaluated. Improved DMPC liposome solubilization is achieved with a hydrophilic (Z)‐end‐group, shorter polystyrene block and lower molecular weight for diblock R‐(Sty)‐b‐(Sty‐alt‐MA)‐T‐Z polymers. Quartz crystal microbalance with dissipation monitoring (QCM‐D) and atomic force microscopy (AFM) revealed that diblock SMA polymers bound to gold as a micellular film, irrespective of the presence of the trithiocarbonate group. SMALPs, however, showed an enhanced gold affinity when terminated by a trithiocarbonate and hydrophilic RAFT (Z)‐end‐group compared to end‐group removed SMALPs, the latter exhibiting nonspecific gold adhesion. These findings offer a new approach in utilizing RAFT end‐groups of nanodisc assembling polymers for label‐free analysis of MPs.

## Graphical abstract

Styrene maleic acid lipid nanoparticles (SMALPs) comprised of diblock styrene maleic acid (SMA) copolymers readily tether to gold surfaces. This binding is enhanced when the diblock SMA is functionalized with a trithiocarbonate and hydrophilic (Z)‐end‐group generated through the reversible addition‐fragmentation chain transfer polymerization process.

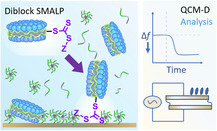

## 1 Introduction

Synthetic polymer nanodiscs are structures that arise from amphipathic copolymers capable of capturing membrane proteins and associated phospholipids into water soluble discs. They can facilitate the purification and investigation of native membrane proteins or protein–protein complexes by replacing the role of detergents typically needed in membrane protein (MP) solubilization.^[^ ^1^, ^2^ ^]^ Polymer nanodiscs have recently also shown potential for diagnostic imaging and the delivery of therapeutics.^[^ ^3^, ^4^ ^]^ The discovery of the first synthetic polymer nanodiscs as an MP reconstitution tool was made by Knowles, Dafforn and Overduin et al.^[^ ^5^ ^]^ where the self‐assembly of poly(styrene‐*co*‐maleic acid) (SMA) into membrane protein encapsulated nanodisc particles was observed upon incubation with biological membranes. The mechanism by which SMALPs form involves the negatively charged carboxylate groups of SMA binding to the hydrophilic surface of the membrane,^[^ ^6^ ^]^ after which the styrene units of the polymer insert between the hydrophobic lipid tail groups of the membrane and polymers proceed to package membrane bundles into thermodynamically favorable nanodiscs.^[^ ^7^, ^8^ ^]^ Styrene maleic acid lipid particles (SMALPs) are optimally formed using SMA copolymers with 2:1 or 3:1 ratios of styrene to maleic acid, which can directly extract a diverse range of membrane proteins, either from native cellular membranes into native nanodiscs or from intermediary reconstituted synthetic membrane systems (such as, liposomes), to yield self‐assembled polymer nanodiscs with 10–30 nm diameter.^[^ ^6^, ^9^ ^]^ Due to their amphipathic nature, SMALPs are able to non‐selectively extract a vast array of membrane components and specific membrane proteins can subsequently be isolated using standard biochemical protein purification techniques, such as affinity chromatography.^[^ ^10^, ^11^ ^]^ SMA can create nanodiscs with size tuneability according to the proportion of polymer and membrane constituents,^[^ ^12^, ^13^ ^]^ allowing for the reconstitution of MPs and MP complexes with varied size and complexity for analysis by techniques, including NMR^[^ ^12^, ^14^ ^]^ and single‐particle cryo‐EM.^[^ ^10^, ^15^, ^16^ ^]^

Reversible addition‐fragmentation chain transfer (RAFT) polymerization is a method of polymer synthesis that offers enhanced sequence control compared to conventional radical polymerization, is compatible with a wide variety of monomer combinations, and allows individual polymer chain lengths to fall within a narrow dispersity (*Đ*) range.^[^ ^17^, ^18^ ^]^ Additionally, each polymer synthesized by RAFT is functionalized by two end‐groups on either end, one at the beginning of the polymer chain known as the *α*‐ or (R)‐end‐group and one at the polymer terminus, the so called *ω*‐ or (Z)‐end‐group.^[^ ^19^ ^]^ These end‐groups can be strategically selected or modified to impart the polymer with a desired chemical functionality and associated properties. Altogether, these features allow RAFT to be applied to many scientific endeavors including bioengineering and nanotechnology.^[^ ^18^, ^20^ ^]^ In the case of SMA and SMALPs, opportunities to modulate polymer sequence, chain length, monomer composition, and end‐group can be exploited by RAFT. Potential benefits include improved nanodisc size homogeneity, unique and precise sequence architecture and SMA conjugation, via the RAFT end‐groups, to a chemical moiety of interest.^[^ ^21^, ^22^, ^23^ ^]^

The additional question of whether the RAFT end‐groups, which terminate each polymer synthesized by RAFT polymerization, can be used to tether polymer nanodiscs to surfaces, such as gold or glass, is of particular interest for the development of label‐free surface‐sensitive analytical methods for the characterization of native membrane proteins. Proteins are often recombinantly expressed or covalently modified in order to insert chemical or biological tags,^[^ ^24^, ^25^ ^]^ which allow them to anchor to surfaces for biophysical analyses including, but not limited to, single molecule fluorescence spectroscopy,^[^ ^26^ ^]^ solution atomic force microscopy (AFM),^[^ ^27^ ^]^ quartz crystal microbalance with dissipation monitoring (QCM‐D)^[^ ^28^, ^29^ ^]^ and surface plasmon resonance (SPR).^[^ ^25^, ^30^ ^]^ Therefore, surface tethering via the protein solubilizing polymer material holds appeal because the protein can forego unnecessary chemical and genetic modification and remain within a native lipid environment that supports a more accurate representation of protein structure and function in the cellular membrane.^[^ ^6^, ^31^ ^]^

**Figure 1:**
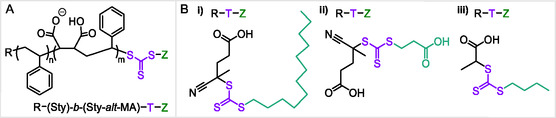
A) Molecular structure of RAFT synthesized diblock SMA and B) RAFT agents selected for SMA synthesis: i) Long hydrophobic RAFT agent 4‐cyano‐4‐[(dodecylsulfanylthiocarbonyl)sulfanyl]pentanoic acid, ii) hydrophilic RAFT agent 4‐((((2‐carboxyethyl)thio)carbonothioyl)thio)‐4‐cyanopentanoic acid, and iii) short hydrophobic RAFT agent 2‐(butylthiocarbonythioythiol) propanoic acid. Trithiocarbonate groups are highlighted in purple and (Z)‐end‐groups are highlighted in green.

We sought to design SMA sequences and SMALPs that could anchor onto gold‐surfaces using RAFT end‐groups attached to each polymer in a proof‐of‐concept study, thereby providing a foundation for the surface‐sensitive analysis of MPs natively reconstituted in SMALPs. The previously reported gold affinity of sulfur‐rich trithiocarbonate moieties, contained in RAFT agents and resulting polymers,^[^ ^32^, ^33^, ^34^ ^]^ is explored in this work for the specific attachment of optimized SMA polymers and related SMALPs encompassing 1,2‐dimyristoyl‐*sn*‐glycero‐3‐phosphocholine (DMPC) phospholipids to a gold surface. Initially, RAFT polymerization was employed to gain a systematic understanding of the relationship between polymer sequence and nanodisc assembly for a range of diblock SMA polymers containing a ≈2:1 Sty:MA monomer ratio. The majority of diblock SMA copolymers in this study incorporated an initial (R)‐end‐group followed by a polystyrene block, a subsequent alternating styrene and maleic acid (Sty‐*alt*‐MA) block and were terminated by a trithiocarbonate (T) moiety attached to the RAFT (Z)‐end‐group of varied hydrophobicity, derived from the RAFT agent selected to control the polymerization. The alternating property of the Sty and MA block is due to both Sty and MAnh monomers in the precursor SMAnh exhibiting low reactivities to growing polymer chains terminated with another unit of itself. MAnh does not self‐polymerise and Sty has a greater propensity to add to chains terminated by a MAnh unit.^[^ ^6^ ^]^ The general R‐(Sty)‐*b*‐(Sty‐*alt*‐MA)‐T‐Z diblock sequence is shown in **Figure** **1**A, alongside the different RAFT agents explored containing long hydrophobic (C_12_H_25_‐), hydrophilic (HOOC‐C_2_H_4_‐) and short hydrophobic (C_4_H_9_‐) (Z)‐end‐groups in Figure 1B (refer to Figure S1, Supporting Information for accompanying CLogP values to denote relative hydrophobicity). In this investigation, the hydrophobicity of the RAFT (Z)‐end group (shown in Figure 1B), the number of polystyrene block repeated units (DP) and the overall molecular weight were varied to determine the optimal diblock SMA sequence architecture for efficient DMPC lipid membrane solubilization. Subsequently, this work explored the ability of various RAFT (Z)‐end‐groups to specifically bind optimized diblock SMA polymers and associated SMALPs to gold surfaces via the sulfur‐rich trithiocarbonate (T) group conjugated to (Z)‐end‐groups of varied chemical structure. We evaluated the effect of varying the (Z)‐end‐group between C_4_H_9_‐ and HOOC‐C_2_H_4_‐ groups as well as the removal of the attached T‐(Z)‐end‐group on the binding of diblock SMA and related SMALPs to gold. Commercial random sequence Lipodisq SMA, attained via a continuous flow of monomers in a 2:1 Sty:MAnh ratio during industrial scale polymerisation, and Lipodisq SMALPs were used for control experiments to assess SMA gold‐affinity in relation to both diblock sequence architecture and end‐group functionalisation.

**Figure 2:**
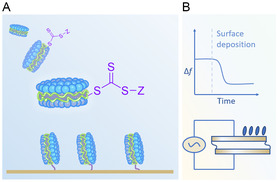
A) Illustration of the expected binding of SMALPs to a gold surface via the solvent exposed trithiocarbonate moiety terminating RAFT synthesized SMA copolymers. The SMALPs depicted are a schematic simplification of their basic structure. B) Representation of a typical quartz crystal microbalance with dissipation monitoring (QCM‐D) measurement showing a negative shift in oscillation frequency (Δ*f*) indicating mass deposition onto a gold‐coated piezoelectric sensor set in motion by an applied alternating current.

A combination of surface‐sensitive analytical methods was employed to quantify and compare gold‐surface deposition. Quartz crystal microbalance with dissipation monitoring (QCM‐D) initially determined the extent of mass deposited by RAFT agents, diblock SMA copolymers and corresponding empty SMALP nanodiscs (comprising 0.2:1 (mol/mol) SMA:DMPC) on a gold surface. Atomic force microscopy (AFM) examined the topography of gold surfaces functionalized with either SMA or SMALPs. Finally, X‐ray photoelectron spectroscopy (XPS) provided complimentary information on the affinity of SMALPs to gold by the detection of phosphorus, originating from phospholipids, on gold surfaces. It was hypothesized that SMA polymers and SMALPs functionalized with the hydrophilic (Z)‐end‐group would better allow the trithiocarbonate functional group to be water accessible rather than trapped within hydrophobic polymer micelles, as represented in **Figure** **2**A. Hydrophilic T‐(Z)‐end‐group terminated SMALPs were therefore expected to have a greater propensity to bind to gold surfaces, as determined by QCM‐D experiments in the manner schematically illustrated in Figure 2B.

## 2 Experimental Section

### 2.1 RAFT Poly(Styrene‐co‐Maleic Anhydride) (SMAnh) Synthesis

For each diblock SMAnh copolymer with a target 2:1 Sty:MAnh monomer unit ratio and a target molecular weight of 12,000, R‐(Sty‐*alt*‐MA)‐*b*‐(Sty)‐T‐Z for **D1** or R‐(Sty)‐*b*‐(Sty‐*alt*‐MA)‐T‐Z for **D2**‐**D4** and **D6**‐**D8** summarized in **Table** **1**, at least two consecutive polymerization reactions were required. For **D1**, the first reaction mixture for block *a* used the [MAnh]:[Sty]:[C_12_RAFT]:[AIBN] ratio of 39:39:1:0.1 and the second reaction mixture used the [polymer]:[Sty]:[AIBN] ratio of 1:39:0.1. For **D2**‐**D10** R‐(Sty)‐*b*‐(Sty‐*alt*‐MA)‐T‐Z diblock SMA polymers, various RAFT agents were selected, C_12_RAFT for **D2** and **D8**, 2‐(butylthiocarbonythioythiol) propanoic acid known as C_4_H_9_‐RAFT for **D3** and **D11** and 4‐((((2‐carboxyethyl)thio)carbonothioyl)thio)‐4‐cyanopentanoic acid (HOOC‐C_2_H_4_‐RAFT) for **D4**, **D5**, **D6**, **D7** and **D10**. The poly(Sty) *a*‐block was initially synthesized using a reaction mixture varied in [Sty]:[RAFT]:[AIBN] ratio with or without a subsequent styrene chain extension polymerization. Another successive polymerization added the *b*‐block containing an alternating 1:1 sequence of Sty and MAnh units by reacting [polymer]:[Sty]:[MAnh]:[AIBN] in the desired ratio. Dioxane solvent was used for each polymerization. Specific details of the synthetic procedure to generate **D2**‐**D10** polymers can be found within the Supporting Information.

**Table 1:** RAFT synthesized diblock SMA copolymer parameter summary using ^1^H NMR and GPC data analysis (*indicates a theoretical value based on precursor parameters). Results from ^31^P NMR and turbidity meter measurements after 24 h incubation of diblock SMA with DMPC LUVs in a 0.2:1 polymer:DMPC lipid ratio are shown.

Diblock copolymer entry	RAFT (Z)‐end‐group	^1^H NMR			GPC			0.2 [polymer]/[DMPC]		
		Sty: MA	Poly (Sty) DP	*M*_n_	*M*_n_	*M*_w_	*Đ*	^31^P NMR nanodisc peak	% Δ Turbidity #1	% Δ Turbidity #2
D1. R‐(Sty‐*alt*‐MA)‐*b*‐(Sty)‐T‐Z	C_12_H_25_	1.7:1	16	9800	12,100	14,600	1.21	Peak	−81.9	−89.4
D2. R‐(Sty)‐*b*‐(Sty‐*alt*‐MA)‐T‐Z	C_12_H_25_	1.7:1	29	13,200	18,100	20,500	1.13	No peak	−55.1	−40.6
D3. R‐(Sty)‐*b*‐(Sty‐*alt*‐MA)‐T‐Z	C_4_H_9_	1.7:1	24	9300	5000	5900	1.18	Peak	−78.1	−83.9
D4. R‐(Sty)‐*b*‐(Sty‐*alt*‐MA)‐T‐Z	C_2_H_4_‐COOH	1.7:1	15	6800	4800	5300	1.11	Peak	−88.2	−91.1
D5. R‐(Sty)‐*b*‐(Sty‐*alt*‐MA)‐T‐Z	C_2_H_4_‐COOH	2.2:1	49	9700	7800	9000	1.15	No peak	−68.7	−65.5
D6. R‐(Sty)‐*b*‐(Sty‐*alt*‐MA)‐T‐Z	C_2_H_4_‐COOH	1.9:1	26	7900	5900	6900	1.18	No peak	−65.6	−69.0
D7. R‐(Sty)‐*b*‐(Sty‐*alt*‐MA)‐T‐Z	C_2_H_4_‐COOH	2.0:1	23	8000	9200	11,000	1.20	Peak	−80.1	−81.8
D8. R‐(Sty)‐*b*‐(Sty‐*alt*‐MA)‐T‐Z	C_12_H_25_	1.7:1	23	7500	7800	9900	1.28	No peak	−14.2	−3.29
D9. R‐(Sty)‐*b*‐(Sty‐*alt*‐MA)‐T‐Z	C_2_H_4_‐COOH	1.5:1	13	7100	5500	6500	1.18	Peak	−97.5	−97.9
D10. R‐(Sty)‐*b*‐(Sty‐*alt*‐MA)‐T‐Z	C_2_H_4_‐COOH	1.7:1	13	5300	4500	5100	1.13	Peak	−97.9	−98.5
D11. R‐(Sty)‐*b*‐(Sty‐*alt*‐MA)‐T‐Z	C_4_H_9_	1.7:1	12	5600	5600	6900	1.23	Peak	−94.7	−94.8
D12. R‐(Sty)‐*b*‐(Sty‐*alt*‐MA)‐T‐H	Removed	1.7:1*	13*	5100*	4000	5100	1.28	Peak	−96.1	−96.1

For each polymerization, the solution was transferred to a Young vessel and degassed by three freeze−pump−thaw cycles before placing the sealed vessel in a 70 °C oil bath for 24 h (or an 85 °C oil bath for **D1**). The viscous solution obtained was diluted in a minimal volume of dioxane and precipitated in either a cosolvent of methanol and hexane for polystyrene precursors or isopropanol for SMAnh copolymers before polymer recovery by vacuum filtration. The redissolution and precipitation procedure was repeated once and the powdered copolymer was dried in a vacuum oven. As is summarized in Table 1, number‐average molar mass (*M*_n_) was determined from ^1^H NMR. Percentage polymer conversion and styrene:maleic anhydride ratios were determined by integrating the crude and pure ^1^H NMR spectra (in acetone‐*d*_6_ or CDCl_3_) for each respective SMAnh synthesis.

### 2.2 Hydrolysis of Poly(Styrene‐co‐Maleic Anhydride) (SMAnh) to Poly(Styrene‐co‐Maleic Acid) SMA

Hydrolysis was undertaken to ring‐open the SMAnh copolymer into SMA in an 2:1 (vol/vol) THF:water solution. Triethylamine (TEA) base was added in a molar ratio of 5.2 × MAnh unit per polymer. This procedure initially dissolved the polymer which was then stirred at 100 °C for 2 h. Hydrolysed polymer was precipitated using 1 M HCl, decanted and then redissolved in minimal ethanol, the precipitation and decantation was repeated once before drying in a fume hood. Hydrolysis of poly(styrene‐*co*‐maleic anhydride) to poly(styrene‐*co*‐maleic acid) using triethylamine (TEA) was confirmed using an Agilent technologies Cary 630 FTIR spectrometer. Diblock SMA was then separated from residual HCl and low molecular weight products by dissolution in Tris‐HCl 20 mm (pH 8.0) and dialysis against 1 L of the same buffer for 24 h with one buffer change. Resulting SMA polymers were freeze‐dried at −80 °C and stored at 4 °C until ready for use. Number‐average molar mass (*M*_n_), weight‐average molar mass (*M*_w_) and dispersity (*Đ* = *M*_w_ */M*_n_) of SMA were obtained from GPC (in DMF) of the hydrolysed product.

### 2.3 Radical‐Induced Reduction to Remove the Trithiocarbonate‐(Z) End‐Group from Diblock SMA

Using diblock SMA **D10** as a starting material, the trithiocarbonate‐(Z)‐RAFT end‐group removal reaction via a radical‐induced reduction process was executed under vacuum sealed conditions. **D10** SMA was reacted with excess benzoyl peroxide initiator and 1‐methyl‐1,4‐cyclohexadiene as a hydrogen donor to give the **D12** analog. Briefly, the poly(styrene‐*co*‐maleic acid (0.02 mmol, 1 equiv.) was combined with 1‐methyl‐1,4‐cyclohexadiene (0.29 mmol, 15 equiv.) and benzoyl peroxide (0.08 mmol, 4 equiv.), dissolved in 5–10 mL dioxane and transferred to a 250 mL Young vessel. The flask was immersed in a 100 °C oil bath following 3 freeze‐vacuum‐thaw cycles to eliminate air. The reaction proceeded for 4 h and was stopped by cooling the vessel down to room temperature. The **D12** product was precipitated dropwise into a hexane/diethyl ether cosolvent and redissolved in acetone/water. The redissolution and precipitation procedure was repeated and the end‐group removed copolymer was dried in a vacuum oven. Resulting SMA was dissolved in 20 mm Tris‐HCl (pH 8.0) and dialysed against 1 L of the same buffer for 24 h, during which the dialysis buffer was exchanged for distilled water. The polymer was then freeze‐dried at −80 °C and refrigerated at 4 °C until ready for use. A combination of UV‐vis analysis and GPC (in DMF) were used to validate the end‐group removal through the disappearance of the absorbance peak at ≈314 nm characteristic of the trithiocarbonate group, while a single peak on the GPC chromatogram proved that unwanted polymer‐polymer recombination was prevented.

### 2.4 Gel Permeation Chromatography (GPC)

Gel Permeation Chromatography (GPC) was performed to measure the molecular weight distribution of all diblock SMA copolymers with a system comprising a Shimadzu LC‐20AT pump, Shimadzu RID‐20A refractive index detector and SPD‐20A UV‐Visible detector. The GPC was equipped with a guard column (WAT054415) and 3 × Waters GPC columns (WAT044238, WAT044226, WAT044235, 300 mm × 7.8 mm). The eluent was filtered dimethylformamide (DMF) containing 0.01 M lithium bromide at 40 °C (flow rate = 1 mL min^−1^). Number average (*M*_n_) and weight average (*M*_w_) molecular weights were evaluated using Shimadzu software. A calibration curve was obtained from poly(methyl methacrylate) (PMMA) standards (Agilent) ranging from 960 to 1,568,000 g mol^−1^. Samples were prefiltered DMF solvent using a 0.2 μm filter to avoid contamination.

### 2.5 Large Unilamellar Liposome (LUV) Extrusion

Lipid stock solutions of 1,2‐dimyristoyl‐*sn*‐glycero‐3‐phosphocholine (DMPC) were prepared by adding 100 μL aliquots of 50 mm DMPC dissolved in chloroform to individual glass test tubes. Lipid mixtures were initially dried by rotating test tubes under a gentle stream of nitrogen gas, then desiccated under vacuum overnight and stored at −20 °C. Before liposome extrusion, frozen tubes containing lipid mixtures were thawed, hydrated with the desired buffer, briefly vortexed, incubated at 37 °C for at least 30 min and vortexed for a further 5 min. Each hydrated lipid mixture (5 mm) was extruded through a polycarbonate membrane of 0.1 μm pore size or 0.2 μm pore size to form large unilamellar vesicles (LUVs) using a mini extruder apparatus (AvantiPolar Lipids, Inc.) mounted on a 40 °C heating block.

### 2.6 Phosphorus Nuclear Magnetic Resonance Spectroscopy (^31^P NMR)

Phosphorus NMR was performed as an initial screening measure to indicate the solubilization of DMPC phospholipids into SMALPs by various diblock SMA copolymers. Extruded DMPC (5 mm) ≈0.1 μm diameter LUV stock solutions were mixed with 2.5 mm polymer (or 3.5 mm for Lipodisq SMA) in deuterated buffer (50 mm Tris‐HCl, 150 mm NaCl, 10% D_2_O, pH 8.00 ± 0.02) in the specified polymer:lipid ratio. For diblock SMA, the 0.2:1 (mol/mol) SMA:DMPC molar ratio was employed and ≈0.28:1 (mol/mol) SMA:DMPC was used for Lipodisq measurements. After mixing the polymer with LUVs, all samples (containing 3.6 mm DMPC) were incubated at 26 °C for at least 24 h. Duplicate^31^P NMR measurements were performed at 25 °C on a Bruker Avance III spectrometer operated at a ^31^P resonance frequency of 162 MHz using a 5 mm triple resonance observe (TBO) probe, according to an adapted method reported by Vargas et al.^[^ ^8^ ^]^ Transients (256) were acquired with an inverse‐gated ^1^H decoupling sequence (zgig30) using an acquisition time of 2.05 s, a sweep width of 64,103 Hz, and a relaxation delay of 6 s. A capillary containing 85% H_3_PO_4_ diluted 1,000‐fold in D_2_O (to ≈15 mm) was used to provide a lock and chemical shift reference (*δ* = 0 ppm). ^31^P NMR spectra were multiplied by an exponential function with a line‐broadening factor of 5.0 Hz before Fourier transformation. Turbidity images were taken of ^31^P NMR samples before and after incubation.

### 2.7 Turbidity Meter Experiments

Turbidity measurements were performed to assess the efficiency of SMALP formation after incubation of diblock SMA with LUVs. The Thermo Scientific Orion AQ4500 Turbidimeter was used in infrared ratio mode with nephelometric turbidity units (NTU). 5 mm DMPC LUV stock solutions extruded through 0.2 μm pore‐sized membranes (840 μL) were added to 1 mm diblock SMA polymer stock solutions (840 μL) to give a 0.2:1 polymer:lipid molar ratio in 50 mm Tris‐HCl (150 mm NaCl, pH = 8.0) buffer. Each mixture was diluted to a final volume of 13.24 mL (0.32 mm DMPC and 0.064 mm SMA polymer). Samples containing DMPC LUVs only and polymer only, at the same concentration, were prepared for comparison. After at least 24 h of incubation, four turbidity measurements were taken for each sample. The percentage difference in (% Δ) turbidity was calculated by subtraction of the mean turbidity of the DMPC LUVs control from the mean turbidity of 0.2:1 polymer:DMPC mixtures, the result of which was then divided by the turbidity of the DMPC LUVs control before multiplication by a factor of 100 to obtain the percentage value. All operations are represented in Equation (1).

(1) % Δ turbidity =100×((mean turbidity of polymer lipid mixture  − mean turbidity of LUV control)/mean turbidity of LUV control)

### 2.8 Transmission Electron Microscopy (TEM)

TEM imaging was conducted to provide particle size information on negatively stained SMALPs and SMA polymer control samples. DMPC LUVs extruded with 0.2 μm polycarbonate pore‐sized filters were combined with SMA dissolved in 50 mm Tris‐HCl buffer (150 mm NaCl, pH = 8.0) in accordance with the designated polymer:lipid molar ratio before further dilution to an overall 1.25 mm DMPC concentration. After at least 24 h of incubation at ≈26 °C, samples were diluted by a factor of 25 before coating TEM grids with sample. For diblock SMA and associated SMALP samples, an ultracentrifugation step was performed (100,000 × g, 90 min, 4 °C) prior to deposition onto grids. Copper grids (formvar/carbon coated, 400 mesh) were plasma glow‐discharged for 20 s to create a hydrophilic surface after which the grids were contacted with a drop of the selected nanodisc mixture. After blotting gently to remove excess sample, the grids were negatively stained with uranyl acetate solution (0.5 % w/v) for 1 min and the grid was dried using a gentle nitrogen stream. Imaging was performed using a FEI Tecnai G2 T20 TWIN TEM instrument operating at 200 kV and equipped with Orius SCD200D wide‐angle CCD camera.

### 2.9 Dynamic Light Scattering (DLS)

After at least 24 h of SMALP incubation, dynamic light scattering (DLS) experiments were performed at 25 °C on a Zetasizer Nano ZS (Malvern Instruments, Worcestershire, UK) to determine the hydrodynamic diameter and purity of samples. For DLS measurements accompanying TEM, 1.25 mm DMPC ≈0.2 μm diameter LUV stock solutions were added to 2.5 mm polymer in a 0.2:1 ratio in Tris‐HCl buffer (alternative SMALP and SMA concentrations were clearly specified when used) prior to SMALP incubation for 24 h at ≈26 °C and ultracentrifugation (100,000 × g, 90 min, 4 °C) to remove non‐solubilised LUVs. DLS measurements were acquired for nanodisc samples 3 consecutive times with 15 runs per measurement. Intensity‐weighted and volume‐weighted size frequency distributions, polydispersity index (PDI) measurements and zeta‐average size (Z‐ave) measurements were generated using ZETASIZER software ver. 7.13 and analyzed using multiple narrow distribution.

### 2.10 Quartz Crystal Microbalance with Dissipation Monitoring (QCM‐D) Experiments

Adsorption and binding of RAFT agents, SMA and related SMALPs onto a gold surface were monitored using the E4 QCM‐D system (Q‐Sense, Sweden). Gold‐coated sensors (QSX 301 Gold (Biolin Scientific)) were cleaned prior to each experiment by soaking in 2% Hellmanex III solution for 10 min, rinsing with water, soaking in a 1:1:3 (vol/vol/vol) hydrogen peroxide:ammonia (20%):water solution for 15 min at 70 °C, rinsing with water and then isopropanol, before UV‐ozone treatment for 20 min. Ozone cleaned sensors were mounted in a Q‐sense E4 system: QE 401 Electronics Unit, QCP 401 Chamber Platform, QSense flow module 401 with four measuring chambers and experiments were carried out between 19 and 22 °C. Changes in frequency (Δ*f*) and dissipation (Δ*D*) signals were recorded using QSoft control software and harmonics from Δ*f*(1) and Δ*D*(1) through to Δ*f*(11) and Δ*D*(11) were recorded to follow the sample adsorption onto the gold surface. Degassed HS PBS buffer (pH = 7.8) was flowed through the QCM‐D system using an Ismatec IPC pump at 200 μL min^−1^ for 10–20 min to obtain a stable baseline and then the pump was stopped before recalibration of stabilized *f* and *D* values to zero and introduction the sample of interest.

Samples examined for their deposition onto gold‐coated sensors were 5 mL solutions of 50 μm RAFT agent (HOOC‐C_2_H_4_‐RAFT or C_4_H_9_‐RAFT), 50 μm diblock SMA (**D10**, **D11** or **D12**) or diblock SMALPs each with DMPC lipids in a 0.2:1 (mol/mol) polymer to lipid ratio (with final SMA concentration standardized to 50 μm) were prepared in HS PBS. Lipodisq SMA and Lipodisq SMALPs using a 1.1:1 (w/w) polymer:lipid ratio were also tested using a higher 70 μm SMA concentration. SMALPs were ultracentrifuged (1,00,000 × g, 90 min, 4 °C) to remove non‐solubilised LUVs prior to diluting the sample to the indicated polymer concentration using a UV‐visible spectrometer (PDA UV/VIS Lambda 265, PerkinElmer) to match the maximal absorbance over a 200–800 nm wavelength to a polymer only standard. The gold‐binding experiment was initiated after 13–15 min to ensure a stable baseline. Either RAFT agent, SMA, or SMALP sample in HS PBS was introduced into the QCM‐D chambers at 50 μL min^−1^ for at least 35 min. The pump was then stopped for ≈15 min and then HS PBS was introduced at 200 μL min^−1^ for 35–40 min before the pump was stopped again for 10 min, after which the experiment was concluded. Measurements for each sample were carried out at least in triplicate to ensure consistent results. The QCM chambers with sensors mounted were cleaned by flowing through water (5 min, 200 μL min^−1^), ethanol (2 min, 200 μL min^−1^), water (5 min, 200 μL min^−1^), 2% Hellmanex III solution (2 min, 200 μL min^−1^) and water (15 min, 200 μL min^−1^), following each experiment.

### 2.11 Quartz Crystal Microbalance with Dissipation Monitoring (QCM‐D) Data Analysis

The raw QCM‐D data was used to calculate the mass coverage (ng cm^−2^) and film thickness (nm) throughout each experiment using the modeling software QTools ver.3 (provided by Q‐sense). Changes in frequency and dissipation data were fitted to the Kelvin‐Voigt viscoelastic model^[^ ^34^ ^]^ described by Equation (2) using the maximum range of harmonics to yield reasonable and consistent mass absorption profiles, ranging between Δ*f*(3) and Δ*D*(3) to Δ*f*(11) and Δ*D*(11). Mass coverage and film thickness were similarly modeled using the Sauerbrey Equation (3) appropriate for rigid films^[^ ^35^ ^]^ using data obtained from the change in frequency of the seventh harmonic (Δ*f*(7)) as the input frequency parameter.

(2) $$G*\;=G\prime \;+iG\prime \prime \;=\mu_{1}+i2\pi f\eta_{1}$$

(3) Δm=−C(Δf/n)

Equation (2) describes the Voigt model for viscoelastic fluids, where G* is the complex shear modulus, *G*′ is the storage modulus and *G*″ is the loss modulus with *μ*, *f* and *η* denoting elasticity, frequency, and shear viscosity coefficient, respectively. This relationship allows for an estimation of the density, thickness and mass of a viscoelastic film bound to the sensor surface. Equation (3) outlines the Sauerbrey equation where Δ*m* is the shift in mass, Δ*f* is the shift in frequency, *C* is the mass sensitivity constant, which is 17.7 ng cm^−2^ Hz^−1^ for a 5 MHz crystal, and *n* is the harmonic number.^[^ ^35^ ^]^ Mean mass coverage and film thickness were calculated for different samples using either duplicate (for RAFT agent gold‐binding) or triplicate (for SMA and SMALP gold‐binding) modeled data from the precise time the pump was stopped after the final buffer wash to report only firmly gold‐bound material. Error bars denoted standard deviation from the mean.

### 2.12 Gold‐Coated Silicon Wafer Fabrication

X‐ray photoelectron spectroscopy (XPS) and atomic force microscope (AFM) experiments required fabrication of gold‐coated silicon wafers. A piece of standard silicon wafer (0.5 mm thickness) was divided into 5 × 5 mm fragments using a tungsten carbide needle to outline each wafer perimeter, then the 5 × 5 mm wafers were broken apart. The surfaces of the wafers were cleaned by placing individual wafers in Eppendorf tubes containing “Decon 90” (5%) solution in water. These tubes were placed in an ultrasonic bath and were sonicated for 30 min at 60˚ C. Wafers were then transferred to clean individual Eppendorf tubes containing Milli‐Q water and sonicated for 10 min. This last step was repeated three times. The fragments were then dried using a stream of nitrogen gas and placed on a parafilm coated glass slide, that was installed into a sputter coater (BAL‐TEC SCD 005) and coated with 20 ± 5 nm of gold (99.99% purity).

### 2.13 X‐Ray Photoelectron Spectroscopy (XPS)

The chemical specificity of diblock SMALP binding to gold was probed by using XPS to detect S‐Au binding for samples which were drop‐casted onto gold‐coated silicon wafers. Diblock SMA polymer solutions (**D10**, **D11** or **D12**) in HS PBS (pH = 7.8) were added to DMPC ≈0.1 μm diameter LUV suspensions in HS PBS to give an overall 0.2:1 polymer to lipid molar ratio (mixtures comprised 1 mm polymer and 5 mm DMPC). While polymer only stock solutions were made up to 1 mm SMA in HS PBS as a control. Polymer‐lipid mixtures were incubated at 26 °C overnight prior to ultracentrifugation (1,00,000 × g, 90 min, 4 °C) and DLS to measure nanodisc size while confirming the removal of residual LUVs and aggregates. A solution of hydrophilic HOOC‐C_2_H_4_‐RAFT agent was made up to 1 mm in HS PBS to determine the chemical specificity of its gold binding and to provide a benchmark for SMALP binding to gold. SMALP and HOOC‐C_2_H_4_‐RAFT samples were drop‐casted in 20 μL volumes onto 5 × 5 mm silicon wafers coated with 20 ± 5 nm gold and samples were allowed to dry in a fume hood before placement in a vacuum desiccator for 30 min with the vacuum pump on and then overnight with the pump off.

A second set of samples containing SMA (**D10** or **D12**) and associated 0.2:1 SMA:DMPC SMALPs were prepared for XPS using the same procedure described earlier, but instead were dissolved in phosphate‐free 50 mm Tris‐HCl buffer (150 mm NaCl, pH = 8.0) for XPS analysis of phosphorus content. These samples were diluted to 0.25 mm as the final polymer concentration. Samples (20 μL volume) were dropped onto UV‐ozone cleaned 5 × 5 mm silicon wafers coated with 20 ± 5 nm gold. After 15 min of deposition time, wafers were washed by their immersion in a reservoir of Tris‐HCl buffer in a petri dish with shaking for 30 min. Buffer was removed and replaced with ultrapure water prior to shaking for another 30 min, removing the water and allowing wafers to dry in a fume hood. Wafers were then placed in a vacuum desiccator for 30 min with the vacuum pump on and for a further 2 h with the pump off. Incorporation of the washing procedure using Tris‐HCl instead of HS PBS buffer enabled the analysis of phosphorus content, and therefore SMALPs, bound to gold. The presence of phosphorus (P 2*p*) was probed on the surface to determine SMALP binding by high‐resolution XPS scans. Quantitative analysis of survey XPS scans revealed the relative carbon content (C 1*s*) bound to gold‐coated washed wafers by calculation of the area under the C 1*s* peak (≈285–286 eV binding energy). The C 1*s* peak area of each sample was averaged from triplicate measurements after subtraction of the mean C 1*s* peak area from a buffer treated gold‐coated (control) wafer.

XPS was performed on a Thermo Scientific Nexsa Surface Analysis System equipped with a hemispherical analyser. The incident radiation was monochromatic Al Kα X‐rays (1486.6 eV) at 72 W (6 mA and 12 kV, 400 μm × 250 μm spot for a 400 μm setting). Survey (wide) and high‐resolution (narrow) scans were recorded at analyser pass energies of 150 and 50 eV and with a step size of 1.0 eV and a dwell time of 10 ms. The base pressure in the analysis chamber was less than 5.0 × 10^−9^ mbar. A low‐energy dual‐beam (ion and electron) flood gun was used to compensate for surface charging. Data processing was carried out using Avantage software (version 5.9931) where the ‘Smart’ background was selected for sulfur (S 2*p*) and phosporus (P 2*p*) curve fitting.

### 2.14 Atomic Force Microscopy (AFM)

AFM was used to map the 3D surface topography of diblock SMA and associated SMALPs bound to the surface of gold‐coated silicon wafers. SMALP samples in HS PBS buffer were prepared from **D10** and **D12** SMA using the same procedure as for XPS experiments. Samples were diluted 4‐fold to give 0.2:1 (mol/mol) SMA:DMPC SMALPs with polymer and lipid concentrations of 0.25 mm SMA and 1.25 mm DMPC, respectively. Separate polymer diblock SMA (0.25 mm) control samples were also prepared. To blot the sample onto the wafer, a 15 μL drop of sample solution was placed onto a gold‐coated silicon wafer (5 × 5 mm width, ≈1 mm thickness, 20 ± 5 nm sputtered gold layer) and incubated for 1 min. The solution was removed from the gold surface using a rapid stream of nitrogen gas. To prevent salt crystallization, a drop (30 μL) of ultrapure water was placed onto the gold wafer surface and was immediately removed using a stream of nitrogen gas.

Another set of SMALP (0.2:1 SMA:DMPC) AFM samples containing 0.25 mm SMA were prepared by adding 30 μL of each sample solution onto individual UV‐ozone cleaned gold‐coated silicon wafers and allowing 15 min of deposition time. Samples were rigorously washed by immersion into a reservoir of HS PBS in a petri dish with shaking for 30 min at ambient temperature. HS PBS was removed and replaced with ultrapure water prior to shaking for another 30 min, removing the water and allowing wafers to air dry in a fume hood. Topographic images of the samples were taken using an AFM (NTEGRA, NT‐MDT, Russia) in semi‐contact mode with simultaneous phase imaging using silicon AFM probes (Tap 300, aluminum reflex coating, ProSciTech, resonance frequency = 300 kHz, force constant = 40 N m^−1^). Resulting datasets were processed, visualized, and analyzed using Nova (NT‐MDT, Russia) software.

## 3 Results and Discussion

### 3.1 Systematic Diblock Copolymer Sequence Optimisation

With the intention to test the ability of RAFT end‐group terminated diblock SMA copolymers to bind to gold via the trithiocarbonate group connecting the RAFT (Z)‐end‐group to the copolymer backbone, a series of diblock copolymers were synthesized with differences in (Z)‐end‐group, sequence, molecular weight, and overall Sty:MA ratio. These diblock SMA polymers were initially screened and optimized for the ability to self‐assemble into SMALPs following addition to DMPC LUVs in a the 0.2:1 (mol/mol) polymer:lipid ratio using ^31^P NMR to confirm nanodisc formation and turbidity meter monitoring to quantify solubilization efficiency. Significant parameters of diblock SMA copolymers are summarized in Table 1, including the RAFT (Z)‐end‐group, Sty:MA ratios determined by ^1^H NMR, degree of polymerization (DP) in the polystyrene block (poly(Sty)), number average molecular weight (*M*_n_) determined by ^1^H NMR, and gel permeation chromatography (GPC) determined *M*_n_, weight average molecular weight (*M*_w_) and dispersity (*Đ*) values. Additionally, results from ^31^P NMR and turbidity meter monitoring after 24 h incubation of SMA with DMPC LUVs at 0.2:1 polymer:lipid (mol/mol) ratio are presented for each polymer in Table 1, while Figure S2, Supporting Information displays supplementary images capturing sample turbidity for 0.2:1 SMA:DMPC SMALPs formed from the library of diblock SMA copolymers. Aside from **D1** (R‐ (Sty‐*alt*‐MA)‐*b*‐(Sty)‐T‐Z), all diblock polymers (**D2**‐**D8**) were prepared with the initial polystyrene block proceeded by the alternating SMA block, giving the sequence arrangement of R‐(Sty)‐*b*‐(Sty‐*alt*‐MA)‐T‐Z. For each diblock SMA structure, with the exception of **D1**, the trithiocarbonate and (Z)‐end‐group was attached to the hydrophilic block of the polymer to encourage trithiocarbonate exposure to the buffered solution and thereby, availability for binding to surfaces.

Differences in the hydrophobic and hydrophilic profile along the SMA polymer chain were affected by both the selection and position of the (Z)‐end‐group. This accounts for why **D1** was the only diblock copolymer with a long hydrophobic (C_12_H_25_) (Z)‐end‐group with the ability to form nanodiscs, evident from an isotropic nanodisc peak at −0.7 ppm in ^31^P NMR data and the 85.7% average reduction in turbidity after addition to DMPC LUVs. The long hydrophobic (Z)‐end‐group of **D1** extends on the hydrophobic polystyrene block whereas for polymers **D2** and **D8**, which did not form SMALPs, the hydrophilic Sty‐*alt*‐MA block is sandwiched between two hydrophobic components, the polystyrene block and the C_12_H_25_ (Z)‐end‐group, giving these amphipathic polymers sequence properties analogous to a triblock copolymer (refer to Table S1, Supporting Information for a visual map of diblock SMA sequences).

Alternatively, **D3** SMA terminated by the short‐hydrophobic (Z)‐end‐group (C_4_H_9_), exhibited nanodisc forming capability in ^31^ P NMR and turbidity measurements with 81% mean reduction in turbidity after addition to DMPC LUVs. Given **D3** has the same approximate Sty:MA ratio (1.7:1) and poly(Sty) DP (23–24) as **D8**, which was similarly synthesized in a two‐step polymerization, these results reflect the impact of different RAFT (Z)‐end‐groups. Thus, a slight change in the hydrophobic chain length of the (Z)‐end‐group within R‐(Sty)‐*b*‐(Sty‐*alt*‐MA)‐T‐Z diblock copolymers can alter the amphipathic sequence of the polymer enough that it affects whether nanodisc assembly can proceed. Also evident from Table 1 is that SMA diblock polymers bearing the hydrophilic C_2_H_4_‐COOH can form nanodiscs for polymers **D4** and **D7** while this was not the case for **D5** an **D6** copolymers with the same terminal (Z)‐end‐group. This variability was unexpected as the hydrophilic (Z)‐end‐group would presumably be the least disruptive to the hydrophilic property of the alternating SMA block to which it is attached. Further analysis of polymer parameters revealed that nanodisc forming polymers (**D4** and **D7**) contained fewer styrene units in their polystyrene block (Table 1) and **D5** and **D6** polymers were synthesized in two steps rather than in three, as an extra polymerization step was performed for some polymers with the objective to add more styrene units to their polystyrene block. Thus, it was concluded that there is a length limit for the polystyrene block within R‐(Sty)‐*b*‐(Sty‐*alt*‐MA)‐T‐Z sequences, above which polymers are not effective nanodisc forming agents.

To maximize the solubilization efficiency of diblock SMA, a shorter 13‐unit polystyrene block was incorporated in two additionally synthesized hydrophilic (Z)‐end‐group terminated R‐(Sty)‐*b*‐(Sty‐*alt*‐MA)‐T‐Z polymer variants, producing **D9** (1.5:1 Sty:MA ratio) and the shorter **D10** (1.7:1 Sty:MA ratio) polymer. Similarly, the 12‐unit polystyrene block was adjoined to **D11** SMA (1.7:1 Sty:MA ratio), an analogous polymer carrying a short‐hydrophobic (C_4_H_9_) (Z)‐end‐group. The act of shortening the polystyrene tail expectedly resulted in an increased mean % turbidity reduction compared with previously screened diblock SMA sequences, these being 97.7% for **D9**, 98.2% for **D10** and 94.8% for **D11** in Table 1.

The superior performance of the hydrophilic terminated **D10** SMA polymer, shown in turbidity monitoring, was reiterated in the ^31^P NMR comparison of Figure S4 (Supporting Information) where **D10** displayed the most intense ≈−1 ppm nanodisc peak. Furthermore, DLS results in Figure S4, Supporting Information supported the effectiveness of both a shorter polystyrene tail and a hydrophilic (Z)‐end‐group as the percentage scattering intensity of the nanodisc peak was between 50% and 60% for **D9** and **D10** in polymer‐lipid mixtures after incubation at the 0.2:1 SMA:DMPC molar ratio. This percentage was markedly higher than that demonstrated by **D4** comprising a hydrophilic (Z)‐end‐group and a slightly longer 15‐unit polystyrene tail at 9.4% nanodisc scattering intensity and short‐hydrophobic (Z)‐end‐group functionalised **D11** (12‐unit polystyrene block) reporting 23.3% nanodisc scattering intensity.

### 3.2 Polymer Modification to Assess the Gold‐Tethering of Trithiocarbonate

To evaluate the impact of the trithiocarbonate moiety and of varied (Z)‐end‐group on diblock SMA and SMALP gold‐surface binding, comparisons were made between diblock SMA and related SMALPs terminated with either the short‐hydrophobic C_4_H_9_ alkyl group (**D11**), the hydrophilic C_2_H_4_‐COOH carboxylic acid group (**D10**) and after the removal of the trithiocarbonate and attached (Z)‐end‐group altogether (**D12**). Cleavage of the trithiocarbonate was achieved by a radical‐induced reduction reaction,^[^ ^36^ ^]^ whereby hydrophilic terminated SMA (**D10**) was combined and reacted with excess benzoyl peroxide initiator and hydrogen donor species 1‐methyl‐1,4‐cyclohexdiene (Figure S5A, Supporting Information) in a degassed Young‐vessel. The success of the trithiocarbonate‐(Z)‐end‐group cleavage reaction was confirmed by UV‐visible spectroscopy where the absorbance peak at *λ*_max_ 315 nm, associated with the trithiocarbonate group of the **D10** precursor, disappeared after its cleavage to form **D12** (refer to Figure S5B, Supporting Information). Gel permeation chromatography (GPC) in DMF revealed a narrow dispersity (*Đ *= 1.28) of the resulting **D12** polymer with a similar molecular weight (*M*_n_ = 4,000, *M*_w_ = 5,100) to the **D10** precursor (*Đ *= 1.13, *M*_n_ = 4,500, *M*_w_ = 5,100). Phosphorus NMR (^31^P NMR) confirmed the formation of an isotropic nanodisc peak between −0.8 and −1.0 ppm after incubation of both hydrophilic **D10** and T‐(Z)‐end‐group removed **D12** SMA with DMPC LUVs at 0.2:1 (mol/mol) SMA:DMPC (Figure S3, Supporting Information). Efficient nanodisc solubilization of DMPC lipid membrane systems before and after diblock SMA T‐(Z)‐end‐group removal was similarly supported in turbidity meter data summarized in Table 1, with mean % turbidity reductions of 98.2% and 96.1% after the incubation of DMPC LUV suspensions with **D10** and **D12** SMA (at 0.2:1 (mol/mol) SMA:DMPC), respectively. Thus, any difference between **D10** and **D12** SMA polymers and associated SMALPs in their surface binding to gold can be directly related to the presence or absence of the trithiocarbonate and (Z)‐end‐group.

### 3.3 Effect of End‐Group Removal on SMALP and SMA Particle Size

**Figure 3:**
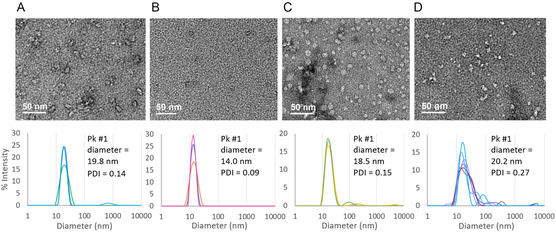
TEM images and accompanying intensity weighted DLS size‐frequency distributions of A) 0.2:1 (mol/mol) SMA:DMPC SMALPs (**D10**), B) **D10** SMA, C) 0.2:1 (mol/mol) SMA:DMPC SMALPs (**D12**) and D) **D12** SMA, each after ultracentrifugation. All DLS measurements used SMA concentrations ranging between 0.1 and 0.25 mm (which were precisely as follows: 0.22 mm for **D10** SMALPs, 0.22 mm for **D10** SMA polymer, 0.25 mm for **D12** SMALPs and 0.1 mm for **D12** SMA polymer). Polydispersity index (PDI) and intensity weighted nanodisc peak size (Pk #1 diameter) are summarized.

The role of the hydrophilic T‐(Z)‐end‐group on the assembly and relative size of SMA micelles and SMALPs was investigated using TEM and DLS measurements in **Figure** **3**. This was done by comparison of **D10** and end‐group removed **D12** SMA polymer samples alongside associated SMALPs comprised of DMPC lipids. DLS particle size‐frequency distributions of **D10** and **D12** (0.2:1 SMA:DMPC) SMALPs revealed close agreement in nanodisc peak diameters of 19.8 nm for **D10** SMALPs in Figure 3A and 18.5 nm for **D12** SMALPs in Figure 3C.

Whereas for **D10** and **D12** SMA polymer samples, there was a marked difference in particle diameter and size dispersity in DLS. The polydispersity indexes (PDIs) were 0.09 for **D10** and 0.27 for **D12** while polymer micelle peak diameters were 14.0 nm and 20.2 nm, respectively. This implies that the C_2_H_4_‐COOH (Z)‐end‐group plays a role in stabilizing free polymer in solution because, after T‐(Z)‐end‐group removal, the less hydrophilic **D12** became more prone to micelle assembly and aggregation. Such a discrepancy in polymer micelle size may affect the mass deposition of **D12** SMA compared to **D10** SMA samples on gold‐coated sensors in the proceeding QCM‐D experiments.

### 3.4 Systematic QCM‐D Analysis of Diblock SMA and SMALP Affinity to Gold

QCM‐D was an exemplary method to assess the impact of T‐(Z)‐end‐groups on SMA and SMALP binding to a gold surface. These experiments systematically measured the mass deposition of RAFT agents, SMA copolymers and corresponding empty SMALP nanodiscs (comprising 0.2:1 (mol/mol) SMA:DMPC lipid), onto a gold surface. Gold‐coated piezoelectric quartz crystal sensors (5 MHz) were mounted in a flow‐pump chamber and the time‐dependent changes in frequency (Δ*f*, Hz) and energy dissipation (Δ*D*, ×10^−6^) of the sensors, oscillating in response to an applied current, were monitored as RAFT agent, polymer or nanodisc solutions were flowed through the chambers (50 μL min^−1^ flow rate) followed by a buffer wash (200 μL min^−1^ flow rate). As mass binds to the sensor surface, the crystal frequency changes proportionally (Δ*m* ∝ −Δ*f*) according to the Sauerbrey model for rigid films (Equation (3)) if the dissipation is small (Δ*D* ≈ 0). In contrast, larger changes in energy dissipation are associated with viscoelastic films which require a viscoelastic model (Equation (2)) to estimate the surface‐adhered mass.^[^ ^35^, ^37^ ^]^ The ability of QCM‐D to act as a highly sensitive nanogram‐scale balance in solution, facilitates a quantitative and systematic comparison of the mass surface‐coverage, thickness and viscoelasticity of gold‐bound material across RAFT agent, polymer and nanodisc samples.

**Figure 4:**
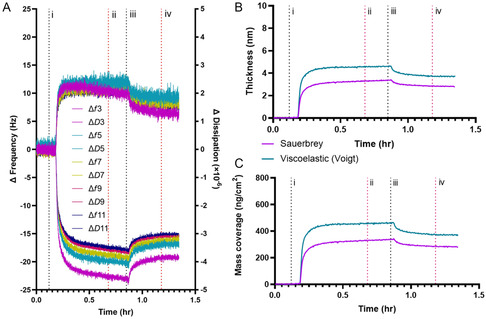
Typical example of a QCM‐D gold binding experiment and modeled data for 50 μm diblock SMA (**D10**). Stages throughout the duration of the measurement are marked: i) polymer addition, ii) no flow (static), iii) buffer wash and iv) no flow (static). In A), time‐dependent changes in frequency (Hz) and dissipation (×10^−6^) readings are shown across harmonics (Δ*f*3–11 and Δ*D*3–11). B) Film thickness (nm) and C) surface mass coverage (ng cm^−2^) versus time are shown as calculated by the Sauerbrey and Kelvin‐Voigt viscoelastic models.

An example of a QCM‐D experiment is seen in **Figure** **4**A, displaying changes in frequency (Hz) and dissipation (×10^−6^) readings over time after an initial HS PBS buffer baseline correction. The harmonics 3 to 11 (Δ*f*3–Δ*f*11 and Δ*D*3–Δ*D*11) are shown following the addition of 50 μm diblock SMA **D10** to the gold‐coated sensor surface. The spanning of Δ*D* values between 1 and 3 (×10^−6^) for all harmonics throughout both polymer flow (i) and final buffer wash stages (iii), along with the spread in frequency between harmonics, indicated the deposition of a viscoelastic polymer film onto the gold surface. Time‐dependent thickness (nm) of the resulting film in Figure 4B and surface mass coverage (ng cm^−2^) in Figure 4C were calculated using the Sauerbrey and Kelvin‐Voigt viscoelastic models.

**Figure 5:**
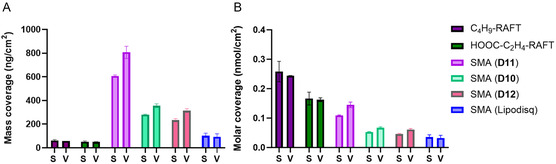
A) The mean mass coverage (ng cm^−2^) and B) mean nanomolar coverage (nmol cm^−2^) of RAFT agents (short‐hydrophobic C_4_H_9_‐RAFT and hydrophilic HOOC‐C_2_H_4_‐RAFT), diblock SMA copolymers (**D11**: short‐hydrophobic T‐(Z)‐end‐group, **D10**: hydrophilic T‐(Z)‐end‐group and **D12**: T‐(Z)‐end‐group removed) and commercial SMA(Lipodisq) deposited onto gold‐coated quartz sensors in QCM‐D experiments according to both Sauerbrey (S) and Kelvin‐Voigt viscoelastic (V) models.

Conversion of data from mass to molar coverage (nmol cm^−2^) was calculated and presented in **Figure** **5**B, which provided additional insight into the number of molecules binding and therefore, the proportional number of sulfur atoms per unit of gold surface area. The observed trend in binding to the gold surface relates strongly to the molecular size and hydrophobicity of the deposited material with the RAFT agents showing the lowest overall film thickness and mass coverage (C_4_H_9_‐RAFT: 61.3 ng cm^−2^ (Sauerbrey), 57.8 ng cm^−2^ (Voigt); HOOC‐C_2_H_4_‐RAFT: 51.2 ng cm^−2^ (Sauerbrey), 49.9 ng cm^−2^ (Voigt)) while simultaneously giving the highest molar coverage (C_4_H_9_‐RAFT: 0.258 nmol cm^−2^ (Sauerbrey), 0.244 nmol cm^−2^ (Voigt); HOOC‐C_2_H_4_‐RAFT: 0.166 nmol cm^−2^ (Sauerbrey), 0.162 nmol cm^−2^ (Voigt)). The more hydrophobic C_4_H_9_‐RAFT showed a higher degree of gold binding in terms of mass and molar coverage, implicating the role of hydrophobic interactions in gold‐surface deposition. The proposed enhancement in gold‐surface binding for more hydrophobic molecules was further supported by the same effect of (Z)‐end‐group hydrophobicity on diblock SMA mass deposition.

C_4_H_9_‐ terminated **D11** claimed the highest mass and molar coverage of all polymers (**D11**: 606.5 ng cm^−2^, 0.109 nmol cm^−2^ (Sauerbrey), 807.3 ng cm^−2^, 0.145 nmol cm^−2^ (Voigt)). Hydrophilic C_2_H_4_‐COOH terminated SMA (**D10**) and its end‐group removed derivative **D12**, produced similar mass and molar coverage (**D10**: 280.5 ng cm^−2^, 0.053 nmol cm^−2^ (Sauerbrey), 355.8 ng cm^−2^, 0.067 nmol cm^−2^ (Voigt); **D12**: 233.5 ng cm^−2^, 0.045 nmol cm^−2^ (Sauerbrey), 312.8 ng cm^−2^, 0.061 nmol cm^−2^ (Voigt)), suggesting that diblock SMA gold binding is not predominately mediated by the trithiocarbonate moiety. The relatively low binding of the commercial Lipodisq sample (101.0 ng cm^−2^, 0.035 nmol cm^−2^ (Sauerbrey), 92.6 ng cm^−2^, 0.032 nmol cm^−2^ (Voigt)), reinforces that the trithiocarbonate group is not necessary for SMA to bind to gold. Lipodisq SMA contains a random rather than diblock sequence and was not polymerised using the RAFT method and therefore contains no trithiocarbonate or (Z)‐end‐group. Lipodisq SMA showed half the degree of gold‐surface deposition compared to RAFT synthesized **D12**, this suggests that the hydrophobic polystyrene block affected a considerable increase in adsorbed mass. The mechanism of SMA binding to gold is likely to involve a network of non‐specific interactions of hydrophobic and hydrophilic groups in copolymers with the gold surface, which similarly accounts for the gold‐adsorption properties of a range of proteins and biomolecules.^[^ ^38^, ^39^ ^]^ A major determinant in the heightened mass deposition observed for species functionalised with hydrophobic (Z)‐end‐groups onto gold, was the greater intermolecular recruitment of molecules into micelles promoted by the hydrophobic effect.

This explanation is supported by the larger SMA polymer micelle size originating from copolymers terminated by short‐hydrophobic (Z)‐end‐groups (**D3** and **D11**) compared to hydrophilic (Z)‐end‐groups (**D10**) in TEM data (Figure S6, Supporting Information) . The similarity in mass deposition estimated by Sauerbrey and Viscoelastic models for each respective RAFT agent and for the commercial Lipodisq SMA on gold, suggests that a rigid film was formed by these less hydrophobic species, which compliments the explanation of a viscoelastic micelle film being formed on gold surfaces for the more hydrophobic diblock SMA.

**Figure 6:**
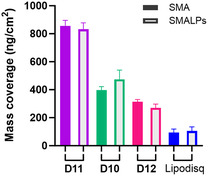
The mean mass coverage (ng cm^−2^) of SMA copolymers (including **D10**, **D11**, **D12** and commercial Lipodisq) and corresponding 0.2:1 (mol/mol) SMA:DMPC diblock SMALPs and 1.1:1 (w/w) Lipodisq:DMPC SMALPs deposited onto gold in QCM‐D experiments according to the Kelvin‐Voigt viscoelastic model. SMA and SMALP samples are denoted by filled and empty columns, respectively.

**Figure 7:**
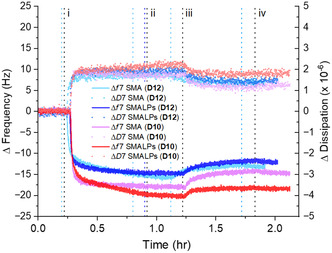
Comparison of representative QCM‐D gold‐binding experiments using **D12** SMA (light blue), **D10** SMA (pink), **D12** SMALPs (dark blue) and **D10** SMALPs (red). SMALP solutions were assembled with 0.2:1 (mol/mol) SMA:DMPC and all samples contained 50 μm SMA. Stages throughout the duration of the measurement are marked: i) SMA or SMALP addition, ii) no flow (static), iii) buffer wash and iv) no flow (static). Standard time‐points for each stage are denoted by a black dotted vertical line and deviations from these time‐points are indicated with light blue and dark blue dotted vertical lines for experiments using **D12** SMA and **D12** SMALPs, respectively. Time‐dependent Δ frequency (Hz) and Δ dissipation (×10^−6^) readings are shown for the 7th harmonic (Δ*f*7 and Δ*D*7).

Formation of SMALPs by reaction of SMA polymers with DMPC LUVs, allows for comparison against SMA polymer alone in binding to gold at an identical polymer concentration. Interestingly, SMALPs displayed a similar average mass surface‐coverage (ng cm^−2^) in QCM‐D to their SMA only counterparts in **Figure** **6** using the viscoelastic model. This suggests a consistent non‐specific interaction between polymer (within polymer micelles or nanodiscs) and gold surfaces across both SMA and SMALP preparations. There was, however, an enhanced mass deposition on the gold surface for trithiocarbonate‐C_2_H_4_‐COOH terminated **D10** SMALPs compared to end‐group cleaved **D12** SMALPs (**D10** SMALPs: 382.9 ng cm^−2^ (Sauerbrey), 481.5 ng cm^−2^ (Voigt); **D12** SMALPs: 211.8 ng cm^−2^ (Sauerbrey), 270.7 ng cm^−2^ (Voigt)). The +125.7 ng cm^−2^ increase in average mass adsorption for **D10** SMALPs compared to **D10** polymer contrasts with the negative −42.1 ng cm^−2^ adhered mass difference observed for end‐group removed **D12** SMALPs compared to **D12** SMA. The difference between these two SMALP samples is further emphasized by the time‐dependent frequency (Δ*f*7) and dissipation (Δ*D*7) plots of individual experiments shown in **Figure** **7** for **D10** and **D12** SMA and corresponding SMALPs.

For the hydrophilic‐trithiocarbonate terminated **D10** SMALPs, a two‐stage frequency curve appears in Figure 7 which was reproduceable across triplicate experiments. This suggests a rapid binding event immediately after SMALP addition, in line with diblock SMA **D10** experimental data, and a second more gradual binding event, evident at the 40–50 min time‐point, thought to result from SMALPs anchoring to gold via the trithiocarbonate group. Complimentary Δ*D* versus Δ*f* plots (refer to Figure S7, Supporting Information) support this interpretation by the depiction of a larger overall change in frequency and dissipation for **D10** SMALPs compared to **D12** SMALPs introduced to a gold surface. Lending additional support this analysis, **D10** SMALPs purified from residual **D10** SMA micelles using ultrafiltration (and adjusted to 50 μm overall polymer concentration) showed a marked increase in overall mass deposition onto gold QCM‐D sensors in comparison to 50 μm **D10** SMA alone (Figure S8, Supporting Information) with **D10** SMALPs covering the gold surface in a more gradual binding process.

Support for direct S‐Au bond formation between the trithiocarbonate in hydrophilic C_2_H_4_‐COOH RAFT agent and the surface of gold‐coated silicon wafers was established in XPS sulfur (S 2*p*) scans in Figure S9 (Supporting Information) after solution drop‐casting. A double peak was revealed in XPS data with maximum intensity at ≈164 eV and a smaller deconvoluted peak at ≈162 eV binding energy, the latter of which is attributable to gold bound sulfur.^[^ ^40^ ^]^ However, this XPS approach was not sensitive enough to detect specific binding between **D10** SMALPS and gold with only a small sulfur signal observed (refer to Figure S9B, Supporting Information), consistent with a reduced overall sulfur percentage within the sample and prevalent non‐specific polymer‐gold interactions. Nevertheless, a separate quantitative XPS analysis of the carbon content remaining on gold‐coated silicon wafers after introducing SMA of SMALP samples, allowing 15 min for surface binding to occur and vigorously washing the surface, mirrored the binding trend of QCM‐D analysis. Diblock SMALPs assembled from **D10** showed the highest mean C 1*s* (≈285–286 eV binding energy) peak area in counts per second × electron volt (6.4 × 10^5^ CPS.eV) followed by **D10** SMA (6.0 × 10^5^ CPS.eV), **D12** SMA (4.8 × 10^5^ CPS.eV) and with **D12** SMALPs showing the smallest mean C 1*s* peak area (4.5 × 10^5^ CPS.eV) and therefore the lowest degree of carbon‐gold deposition (refer to Figure S10, Supporting Information).

The observed difference in gold affinity between SMALPs with and without the hydrophilic T‐(Z)‐end‐group (**D10** and **D12**), which was not found to the same extent for SMA only samples, may be due to nanodiscs having a distinct 3‐dimensional morphology which favors different water exposed moieties compared to polymer only micelles. Accordingly, the trithiocarbonate and hydrophilic (Z)‐end‐groups may protrude outwards, becoming more exposed and sterically available to selectively bind to gold in SMALPs. Alternatively, the apparent similarity in gold affinity of **D10** and **D12** SMA may disguise a lower inherent gold affinity in the T‐(Z)‐end‐group removed **D12** SMA which is partially offset by its larger micelle mass associated in the binding.

### 3.5 Atomic Force Microscopy Investigations of Diblock SMALPs Bound to Gold Surfaces

Atomic force microscopy (AFM) visualization of the surface topography of gold‐bound SMA and SMALPs offered complimentary information on their gold‐binding behavior. The potential coexistence of SMA (**D10**) polymer micelles and SMALPs (**D10**) on the gold‐surface in QCM‐D studies was supported by AFM topographical images of samples deposited onto gold‐coated silicon wafers. The smooth surface of the gold‐coated wafers prior to sample deposition was confirmed in Figure S11, Supporting Information. As is shown in Figure S12, Supporting Information, when diblock SMA (**D10**) polymer was blotted onto the gold surface, a particle size ranging up to ≈20 nm was observed, according to the z‐height range of the scans. The largest particle in the area examined was found to be 22–24 nm using cross‐sectional analysis of **D10** SMA on gold, while the majority of particles, below ≈10 nm, indicated the deposition of polymer micelles onto gold. The *z*‐height parameter provided the metric for particle size due to its superior resolution compared to the lateral *x*‐ and *y*‐ dimensional resolution, which was limited by the larger diameter of the AFM scanning tip. However, a ±5 nm error in *z*‐height can be assumed due to effects of vibrational noise on these data.

**Figure 8:**
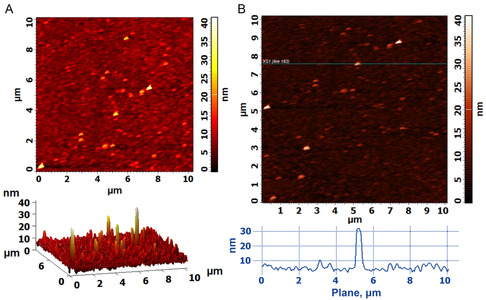
AFM images of 0.2:1 (mol/mol) SMA:DMPC SMALPs (**D10**) added onto a gold‐coated silicon wafer and washed rigorously with buffer and ultrapure water. A) 2D *z*‐height contrast topographical image and accompanying 3D representation (below) and, B) 2D *z*‐height contrast topographical image with an intersecting plane for which a cross‐section plot is given (below).

Conversely, samples of **D10** SMALPs containing DMPC were deposited on gold‐coated silicon wafers and washed thoroughly to simulate QCM‐D experiments to remove any weakly bound material. Two particle size‐populations could be distinguished for **D10** SMALPs in **Figure** **8**A,B. The smaller of these particles has a z‐height of less than ≈15 nm in agreement with the size range expected for SMA micelles determined from the cross‐section plot of Figure 8A. The larger particles in the SMALP **D10** sample showed a *z*‐height of >20 nm in Figure 8A,B, a size population beyond that detected for SMA alone. This larger particle size agreed with TEM images and DLS size frequency distributions for **D10** SMALP samples and implied that a portion of SMALP particles were surface bound with the nanodisc rim‐edge against the surface, giving the z‐height consistent with the nanodisc diameter rather than the (≈5 nm) thickness of the lipid bilayer. These AFM findings are consistent with the hypothesis previously proposed for **D10** SMA and SMALP gold binding in relation to QCM‐D data, whereby a viscoelastic film of SMA copolymer micelles, free polymer and SMALPs initially adhere to the gold surface. A second slower surface binding event follows for the HOOC‐C_2_H_4_‐trithiocarbonate terminated SMALPs (**D10**) mediated by the hydrophilic T‐(Z)‐end‐group.

As was mentioned for TEM and DLS analyses, AFM images were unable to clearly differentiate between T‐(Z)‐end‐group removed (**D12**) SMA and resulting (0.2:1 SMA:DMPC) SMALPs on the basis of size and therefore z‐height was less informative in images for end‐group removed samples. Topographical images, contrasted by z‐height, of **D12** SMA polymer blotted onto gold showed particles ranging up to ≈40 nm according to the z‐height scale and cross‐section plots in Figure S13, Supporting Information. An overlapping particle size population of ≈10–45 nm bound to gold was observed for z‐height contrasted topographical images of **D12** SMALPs (in Figure S14, Supporting Information) which were deposited on gold‐coated silicon wafers and washed thoroughly to simulate QCM‐D experiments. The discrete particles bound to gold were expected to comprise a combination of micelles and **D12** SMALPs non‐specifically adhering to gold.

**Figure 9:**
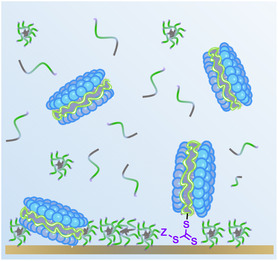
An illustration representing a model for gold surface binding of 0.2:1 (mol/mol) SMA:DMPC SMALPs (**D10**). Diblock SMA micelles and SMALPs are depicted as saturating the gold surface and a proportion of SMALPs (**D10**) were thought to specifically bind to uncovered sites in a slower process via the solvent exposed trithiocarbonate moiety.

The presence of nanodiscs on the surface of washed gold‐coated silicon wafers was qualitatively determined by XPS. Phosphorus scans shown in Figure S15(Supporting Information)  depict a weakly emerging phosphorus 2*p* peak at ≈134 eV maximum binding energy, indirectly signifying the presence of nanodiscs adhered to the gold surface for SMALP samples arising from both hydrophilic‐trithiocarbonate terminated **D10** and end‐group removed **D12**, both prepared in phosphate‐free Tris‐HCl buffer. These findings highlight that non‐specific binding dominates the interaction of diblock SMALPs with gold surfaces and, taken together with QCM‐D and AFM data, support the binding model proposed and illustrated in **Figure** **9**.

In this explanation, a viscoelastic film of SMA copolymer micelles, free polymer and SMALPs non‐specifically adhere to the gold surface. A second more gradual surface binding transpires for HOOC‐C_2_H_4_‐trithiocarbonate terminated SMALPs (**D10**) in a process mediated by the hydrophilic‐(Z)‐trithiocarbonate end‐group moiety affinity to gold. To circumvent non‐specific binding in polymer nanodisc tethering to gold surfaces in the future, alternative polymer nanodisc materials without intrinsic gold‐binding properties, in conjunction with RAFT polymerization, may be developed and implemented in biological membrane research to maximally benefit from the specific binding between gold and sulfur‐rich RAFT end‐groups. Moreover, post polymerization modifications may enhance the tethering of polymers bearing RAFT end‐groups onto a gold surface. For instance, a nucleophilic cleavage reaction to convert the T‐(Z)‐end‐group into a terminal thiol group (‐SH) may generate polymers and associated polymer‐lipid nanodiscs with high SH‐gold affinity.^[^ ^41^ ^]^ Thiols have a widely established affinity to gold and are simple to generate from RAFT polymers,^[^ ^34^ ^]^ thus a comparison between the gold‐tethering behavior of trithiocarbonate‐(Z)‐end‐group and thiol terminated polymer nanodiscs would be beneficial for selecting polymers for prospective membrane protein studies.

## 4 Conclusions

A library of diblock copolymers with the R‐(Sty)‐*b*‐(Sty‐*alt*‐MA)‐T‐Z sequence arrangement were synthesized using RAFT‐mediated polymerization with varied (Z)‐end‐group hydrophobicity, polystyrene block length and molecular weight to determine the optimal diblock SMA structure for liposome solubilization. The diblock architecture of SMA copolymers was selected to impart strict sequence control, low dispersity and the ability to place of the T‐(Z)‐end‐group on the hydrophilic polymer chain terminus, thereby encouraging the availability of the trithiocarbonate for binding activity in aqueous solution. A correlation between improved DMPC liposome solubilization efficiency and a more hydrophilic (Z)‐end‐group, shorter hydrophobic polystyrene block and a lower overall molecular weight for 2:1 Sty:MA diblock R‐(Sty)‐*b*‐(Sty‐*alt*‐MA)‐T‐Z polymers was determined. Furthermore, the ability of trithiocarbonate linked RAFT end‐groups to specifically bind optimized styrene maleic acid lipid nanoparticles (SMALPs) to gold surfaces was explored. Systematic QCM‐D experiments revealed that diblock SMA binds to gold sensors in the form of a viscoelastic film, irrespective of the presence of the trithiocarbonate functional group. SMALPs derived from these polymers did, however, show additional gold affinity dependent on their termination by a hydrophilic trithiocarbonate‐C_2_H_4_‐COOH RAFT end‐group (**D10**), in a slower binding event compared to that of free polymer.

AFM images provided additional insight into the morphology and gold‐surface topography of hydrophilic diblock SMA and SMALPs samples. Both hydrophilic diblock polymer (**D10**) micelles and corresponding SMALPs bound to gold surfaces due to non‐specific interactions. A proportion of hydrophilic terminated (**D10**) SMALPs were thought to participate in additional specific binding to gold surfaces via the aqueous solution exposed trithiocarbonate group modulated by the hydrophilic (Z)‐end‐group. Thus, future studies would benefit from the selection of nanodisc assembling polymers with weak non‐specific affinity for gold to unambiguously determine the extent to which trithiocarbonate and sulfur‐rich RAFT end‐groups can participate in tethering polymer nanodiscs and natively encapsulated membrane proteins to gold interfaces. Controlled 2:1 Sty:MA SMA copolymers comprising a periodic rather than diblock sequence were obtained using iterative RAFT‐mediated polymerization by Cunningham et al.,^[^ ^42^ ^]^ offering an alternative SMA sequence which, as was demonstrated for commercial Lipodisq SMA, may exhibit a lower intrinsic affinity to gold due to the absence of a polystyrene block. Alternatively, an expanding toolbox of amphiphilic polymers with diverse monomer combinations have shown the ability to assemble polymer‐lipid nanodiscs providing the opportunity to further optimize the gold‐affinity demonstrated for trithiocarbonates of RAFT (Z)‐end‐groups and their sulfur containing derivates. Polymer nanodiscs able to tether to gold via the polymer belt expand the prospects for surface‐sensitive analysis label‐free MPs.

## Supplementary Information

The authors have cited additional figures and data within the supplementary Information.

## Conflict of Interest

The authors declare no conflict of interest.

## Author Contributions

**Michelle D. Farrelly**: methodology, investigation, formal analysis, data curation, validation, visualization, conceptualization, writing—original draft and writing—review & editing. **Denis Korneev**: methodology, investigation, formal analysis, validation. **Lisandra L. Martin**: Supervision, conceptualisation, project administration, resources, funding acquisition, writing—review & editing. **San H. Thang**: Supervision, conceptualisation, project administration, resources, funding acquisition, writing—review & editing.

## Supporting information

Supplementary Material

## References

[1] T. Ravula , N. Z. Hardin , G. M. Di Mauro , A. Ramamoorthy , Eur. Polym. J. 2018, 108, 597. 31105326 10.1016/j.eurpolymj.2018.09.048 PMC6516473

[2] D. J. K. Swainsbury , S. Scheidelaar , N. Foster , R. van Grondelle , J. A. Killian , M. R. Jones , Biochim. Biophys. Acta, Biomembr. 2017, 1859, 2133. 28751090 10.1016/j.bbamem.2017.07.011 PMC5593810

[3] M. Tanaka , A. Hosotani , T. Mukai , J. Labelled Compd. Radiopharm. 2018, 61, 857. 10.1002/jlcr.3668 29972867

[4] I. Noh , Z. Guo , J. Zhou , W. Gao , R. H. Fang , L. Zhang , ACS Nano 2023, 17, 1120. 10.1021/acsnano.2c08360 PMC10225015 36441916

[5] T. J. Knowles , R. Finka , C. Smith , Y. Lin , T. Dafforn , M. Overduin , J. Am. Chem. Soc. 2009, 131, 7484. 19449872 10.1021/ja810046q

[6] J. M. Dorr , S. Scheidelaar , M. C. Koorengevel , J. J. Dominguez , M. Schafer , C. A. van Walree , J. A. Killian , Eur. Biophys. J. 2016, 45, 3. 26639665 10.1007/s00249-015-1093-y PMC4698303

[7] S. C. L. Hall , C. Tognoloni , G. J. Price , B. Klumperman , K. J. Edler , T. R. Dafforn , T. Arnold , Biomacromolecules 2018, 19, 761. 29272585 10.1021/acs.biomac.7b01539

[8] C. Vargas , R. C. Arenas , E. Frotscher , S. Keller , Nanoscale 2015, 7, 20685. 26599076 10.1039/c5nr06353a

[9] S. Scheidelaar , M. C. Koorengevel , J. D. Pardo , J. D. Meeldijk , E. Breukink , J. A. Killian , Biophys. J. 2015, 108, 279. 25606677 10.1016/j.bpj.2014.11.3464 PMC4302193

[10] S. Gulati , M. Jamshad , T. J. Knowles , K. A. Morrison , R. Downing , N. Cant , R. Collins , J. B. Koenderink , R. C. Ford , M. Overduin , I. D. Kerr , T. R. Dafforn , A. J. Rothnie , Biochem. J. 2014, 461, 269. 24758594 10.1042/BJ20131477

[11] G. Walker , C. Brown , X. Ge , S. Kumar , M. D. Muzumdar , K. Gupta , M. Bhattacharyya , Nat. Nanotechnol. 2024, 19, 85. 38012273 10.1038/s41565-023-01547-4 PMC10981947

[12] A. F. Craig , E. E. Clark , I. D. Sahu , R. Zhang , N. D. Frantz , M. S. Al‐Abdul‐Wahid , C. Dabney‐Smith , D. Konkolewicz , G. A. Lorigan , Biochim. Biophys. Acta, Biomembr. 2016, 1858, 2931. 10.1016/j.bbamem.2016.08.004 27539205

[13] J. , S. H. Park , S. J. Opella , Biophys. J. 2018, 115, 22. 29914645 10.1016/j.bpj.2018.05.024 PMC6035293

[14] L. Zhu , H. Zhao , Y. Wang , C. Yu , J. Liu , L. Li , Z. Li , J. Zhang , H. Dai , J. Wang , L. Zhu , PeerJ 2022, 10, e13381. 35529497 10.7717/peerj.13381 PMC9074879

[15] C. Sun , S. Benlekbir , P. Venkatakrishnan , Y. Wang , S. Hong , J. Hosler , E. Tajkhorshid , J. L. Rubinstein , R. B. Gennis , Nature 2018, 557, 123. 29695868 10.1038/s41586-018-0061-y PMC6004266

[16] M. Peplow , ACS Cent. Sci. 2020, 6, 1274. 32875068 10.1021/acscentsci.0c01048 PMC7453411

[17] J. Chiefari , Y. K. Chong , F. Ercole , J. Krstina , J. Jeffery , T. P. T. Le , R. T. A. Mayadunne , G. F. Meijs , C. L. Moad , G. Moad , E. Rizzardo , S. H. Thang , Macromolecules 1998, 31, 5559.

[18] G. Moad , E. Rizzardo , S. H. Thang , Chem. Asian J. 2013, 8, 1634. 23606667 10.1002/asia.201300262

[19] J. Vandenbergh , T. Junkers , Macromolecules 2014, 47, 5051.

[20] M. L. Coote , E. H. Krenske , E. I. Izgorodina , Macromol. Rapid Commun. 2006, 27, 473.

[21] A. A. A. Smith , H. E. Autzen , T. Laursen , V. Wu , M. Yen , A. Hall , S. D. Hansen , Y. Cheng , T. Xu , Biomacromolecules 2017, 18, 3706. 28934548 10.1021/acs.biomac.7b01136

[22] G. M. Neville , K. J. Edler , G. J. Price , Nanoscale 2022, 14, 5689. 35315461 10.1039/d1nr07230g

[23] L. E. Ball , M. P. Smith , B. Klumperman , Polym. Chem. 2025, 16, 1019. 39831173 10.1039/d4py01227e PMC11740854

[24] D. L. Johnson , L. L. Martin , J. Am. Chem. Soc. 2005, 127, 2018.15713059

[25] M. Trahey , M. J. Li , H. Kwon , E. L. Woodahl , W. D. McClary , W. M. Atkins , Curr. Protoc. Protein Sci. 2015, 81, 29.13.1. 10.1002/0471140864.ps2913s81 PMC4533999 26237675

[26] R. Lamichhane , J. J. Liu , G. Pljevaljcic , K. L. White , E. van der Schans , V. Katritch , R. C. Stevens , K. Wuthrich , D. P. Millar , Proc. Natl. Acad. Sci. U. S. A. 2015, 112, 14254. 26578769 10.1073/pnas.1519626112 PMC4655547

[27] A. M. Bronder , A. Bieker , S. Elter , M. Etzkorn , D. Haussinger , F. Oesterhelt , Biophys. J. 2016, 111, 1925. 27806274 10.1016/j.bpj.2016.08.051 PMC5103026

[28] E. T. Harrison , Y. C. Wang , L. Carter , D. G. Castner , Biointerphases 2020, 15, 021002. 32168986 10.1116/1.5142560 PMC7069763

[29] M. Wadsäter , T. Laursen , A. Singha , N. S. Hatzakis , D. Stamou , R. Barker , K. Mortensen , R. Feidenhans'l , B. L. Møller , M. Cárdenas , J. Biol. Chem. 2012, 287, 34596. 22891242 10.1074/jbc.M112.400085 PMC3464565

[30] A. Das , J. Zhao , G. C. Schatz , S. G. Sligar , R. P. Van Duyne , Anal. Chem. 2009, 81, 3754. 19364136 10.1021/ac802612z PMC4757437

[31] M. D. Farrelly , L. L. Martin , S. H. Thang , Chem. Eur. J. 2021, 27, 12922. 34180107 10.1002/chem.202101572

[32] A. S. Duwez , P. Guillet , C. Colard , J. Gohy , C. Fustin , Macromolecules 2006, 39, 2729.

[33] C.‐A. Fustin , A.‐S. Duwez , J. Electron Spectrosc. Relat. Phenom. 2009, 172, 104.

[34] S. Slavin , A. H. Soeriyadi , L. Voorhaar , M. R. Whittaker , C. R. Becer , C. Boyer , T. P. Davis , D. M. Haddleton , J. Soft Matter 2012, 8, 118.

[35] A. D. Easley , T. Ma , C. I. Eneh , J. Yun , R. M. Thakur , J. L. Lutkenhaus , J. Polym. Sci. 2021, 60, 1090.

[36] Y. K. Chong , G. Moad , E. Rizzardo , S. H. Thang , Macromolecules 2007, 40, 4446.

[37] I. Reviakine , D. Johannsmann , R. P. Richter , Anal. Chem. 2011, 83, 8838. 21939220 10.1021/ac201778h

[38] E. E. Ferapontova , V. G. Grigorenko , A. M. Egorov , T. Börchers , T. Ruzgas , L. Gorton , Biosens. Bioelectron. 2001, 16, 147. 11339993 10.1016/s0956-5663(01)00134-8

[39] O. Cohavi , D. Reichmann , R. Abramovich , A. B. Tesler , G. Bellapadrona , D. B. Kokh , R. C. Wade , A. Vaskevich , I. Rubinstein , G. Schreiber , Chem. Eur. J. 2011, 17, 1327. 21243701 10.1002/chem.201001781

[40] D. G. Castner , K. Hinds , D. W. Grainger , Langmuir 1996, 12, 5083.

[41] P. J. Roth , C. Boyer , A. B. Lowe , T. P. Davis , Macromol. Rapid Commun. 2011, 32, 1123. 21567648 10.1002/marc.201100127

[42] R. D. Cunningham , A. H. Kopf , B. O. W. Elenbaas , B. B. P. Staal , R. Pfukwa , J. A. Killian , B. Klumperman , Biomacromolecules 2020, 21, 3287. 32672942 10.1021/acs.biomac.0c00736

