# Supplementary material for: Tethering Efficiency of Reversible Addition‐Fragmentation Chain Transfer‐Synthesized Styrene Maleic Acid Polymers and Associated Styrene Maleic Acid Lipid Nanoparticles on Gold Surfaces
Source: Chempluschem. 2025 Apr 17;90(7):e202500173. doi: 10.1002/cplu.202500173 (PMC12261043; doi:10.1002/cplu.202500173)
Supplement: Supplementary file 1 — Supplementary Material [file CPLU-90-e202500173-s001.pdf]

Supplementary Information

Tethering Efficiency of RAFT-Synthesised SMA Polymers and Associated SMALPs on Gold Surfaces

Michelle D Farrelly[a], Denis Korneev[b][c], Lisandra L Martin[a]* and San H Thang[a]*

[a] School of Chemistry, Monash University Clayton, VIC 3800, Australia

[b] Ramaciotti Centre for Cryo-Electron Microscopy, Monash University Clayton, VIC 3800, Australia

[c] School of BioSciences and Bio21 Molecular Sciences and Biotechnology Institute, The University of Melbourne, Parkville, VIC 3010, Australia

Corresponding Authors: Lisa.Martin@monash.edu, San.Thang@monash.edu

Contents

Materials and Methods S3

RAFT Polymerisation of Diblock SMAnh S3

Supplementary Data: Optimisation of Diblock SMA for Efficient LUV Solubilisation S5

Figure S1. RAFT agents selected for SMA synthesis S5

Figure S2. Turbidity observations for all SMALP samples S5

Figure S3. ^{31}P NMR data from equilibrated 0.2:1 SMA:DMPC samples S6

Figure S4. Intensity weighted DLS comparison and ^{31}P NMR spectra for SMALP samples S6

Figure S5. Radical-induced reduction reaction scheme and UV-vis confirmation of T-(Z)-end-group removal to form **D12** SMA S7

Figure S6. TEM images of SMA micelles and corresponding SMALPs S7

Table S1. Sequence design for diblock SMA copolymer library S8

Supplementary Data: Gold Tethering Studies of SMALPs S9

Figure S7. QCM-D plots of ΔD7(x 10^{-6}) versus Δf7(Hz) for SMA and corresponding SMALPs S9

Figure S8. QCM-D gold-binding experiments using **D10** SMA and purified **D10** SMALPs S10

Figure S9. XPS sulfur scans of hydrophilic RAFT agent and **D10** SMALPs blotted on gold S11

Figure S10. XPS of carbon content bound to gold coated silicon wafers after deposition and washing of SMA and corresponding SMALPs on the gold surface S11

Figure S11. AFM images of a gold coated silicon wafer (control) S12

Figure S12. AFM images of diblock SMA (**D10**) blotted onto a gold coated silicon wafer S12

Figure S13. AFM images of diblock SMA (**D12**) blotted onto a gold coated silicon wafer S13

Figure S14. AFM images of **D12** SMALPs deposited on a gold coated silicon wafer S13

Figure S15. XPS phosphorus scans of gold coated silicon wafers after desposition and washing of SMA and SMALPs on the gold surface S14

Materials and Methods

Styrene (99%, Merck, Germany) was purified by passing through an alumina column to remove inhibitor. Azobisisobutyronitrile (AIBN) initiator was purchased from Wako Pure Chemical Industries (Japan) and benzoyl peroxide was purchased from Sigma-Aldrich (USA). Maleic anhydride (99%, Sigma-Aldrich), triethylamine (≥99%, Sigma-Aldrich) and 1-methyl-1,4-cyclohexadiene (≥96%, stabilized, Sigma-Aldrich) were used as received. 4-Cyano-4-[(dodecylsulfanylthiocarbonyl)sulfanyl] pentanoic acid (≥99%, C_{12}RAFT), 2-(butylthiocarbonythioythiol) propanoic acid (95%) and 4-((((2-carboxyethyl)thio) carbonothioyl)thio)-4-cyanopentanoic acid (95%) were purchased from Boron Molecular (Australia). Hellmanex ™ III cleaning concentrate was purchased from Sigma-Aldrich. Other solvents used were analytical or high-performance liquid chromatography (HPLC) grade. Water used was ultrapure (Sartorius or Merck Millipore). Tris (hydroxymethyl) aminomethane hydrochloride (TRIS-HCl) buffer comprised trizma® base (≥99.9%, Sigma) adjusted to the pH of 8.00 ± 0.02 by dropwise addition of ~0.1 M HCl in the presence of the desired NaCl concentration. High salt phosphate-buffered saline (HS PBS) was prepared with potassium phosphate monobasic (99% KH_2PO_4, Sigma), potassium phosphate dibasic (98% K_2HPO_4, Sigma-Aldrich) and 100 mM sodium chloride (NaCl, Sigma) before adjusting the pH to 7.40 ± 0.02 with either ~0.1 M HCl or ~0.1 M NaOH. Lipodisq® 2:1 styrene:maleic anhydride copolymer was purchased from Sigma-Aldrich. The phospholipid 1,2-dimyristoyl-*sn*-glycero-3-phosphocholine (DMPC) was purchased from Avanti® Polar Lipids (USA).

Q-sense® QCM-D sensors used were QSX 301 Gold (Biolin Scientific, Sweden) and AFM probes were silicon AFM probes Tap 300 (Aluminium reflex coating) from ProSciTech (Australia). ^{1}H NMR spectroscopy was performed using a Bruker AVIII 400 MHz spectrometer and infrared spectroscopy was performed with an Agilent technologies Cary 630 FTIR spectrometer. Ultracentrifugation was performed using an Optima™ TLX ultracentrifuge and samples were loaded in 1 mL open-top thick-walled polycarbonate tubes (Beckman Coulter, USA).

RAFT Polymerisation of Diblock SMAnh

For each diblock SMAnh copolymer with a target 2:1 Sty:MAnh monomer unit ratio and a target molecular weight of 12,000, R-(Sty-*alt*-MA)-*b*-(Sty)-T-Z for **D1** or R-(Sty)-*b*-(Sty-*alt*-MA)-T-Z for **D2**-**D4** and **D6**-**D8** summarised in Table 1, at least two consecutive polymerisation reactions were required. For **D1**, the first reaction mixture for block *a* used the [MAnh]:[Sty]:[C_{12}RAFT]:[AIBN] ratio of 39:39:1:0.1 and the second reaction mixture used the [polymer]:[Sty]:[AIBN] ratio of 1:39:0.1. For **D2**-**D4** and **D6**-**D8,** the poly(Sty) *a*-block was initially synthesised using a [Sty]:[RAFT]:[AIBN] ratio of 39-59:1:0.1, depending on the target poly(Sty) chain length, with an additional styrene chain extension polymerisation using a [polymer]:[Sty]:[AIBN] ratio of 1:20-59:0.1 for **D2** and **D6** polymers to compensate for the low % conversion typical of this reaction. The second polymerisation to add the alternating *b*-block used a [polymer]:[Sty]:[MAnh]:[AIBN] reaction mixture of 1:39:39:0.1. Diblock copolymer **D5**, with target Sty:MAnh ratio = 2.5:1 and target M_n = 14,000, used a [Sty]:[HOOC-C_2H_4-RAFT]:[AIBN] ratio of 1:59:0.1 for the first polymerisation with a further Sty chain extension polymerisation performed in a 1:59:0.1 [polymer]:[Sty]:[AIBN] ratio before the final alternating *b*-block synthesis in a 1:39:39:0.1 [polymer]:[MAnh]:[Sty]:[AIBN] ratio.

Diblock copolymers **D9** and **D10**, containing a markedly smaller *a*-block poly(Sty) component, utilised the hydrophilic HOOC-C_2H_4-RAFT agent with target monomer ratios of 1.5:1 Sty:MAnh and 2:1 Sty:MAnh and target molecular weights of 10,200 and 6,100, respectively. The equivalent *a*-block (poly(Sty)) reaction was used for both **D9** and **D10**, involving a [Sty]:[HOOC-C_2H_4-RAFT]:[AIBN] ratio of 20:1:0.1. For **D9**, the alternating *b*-block used a [polymer]:[Sty]:[MAnh]:[AIBN] reaction mixture of 1:40:40:0.1 and for **D10**, the alternating *b*-block used a [polymer]:[Sty]:[MAnh]:[AIBN] reaction mixture of 1:20:20:0.1. **D11** (target molecular weight = 6,100), was synthesised using the same ratio of monomers,

RAFT agent and initiator as **D10** for consecutive blocks, but instead utilising the short hydrophobic C_4H_9-RAFT agent. Dioxane solvent was used for each polymerisation.

<u>Supplementary Data: Optimisation of Diblock SMA for Efficient LUV Solubilisation</u>

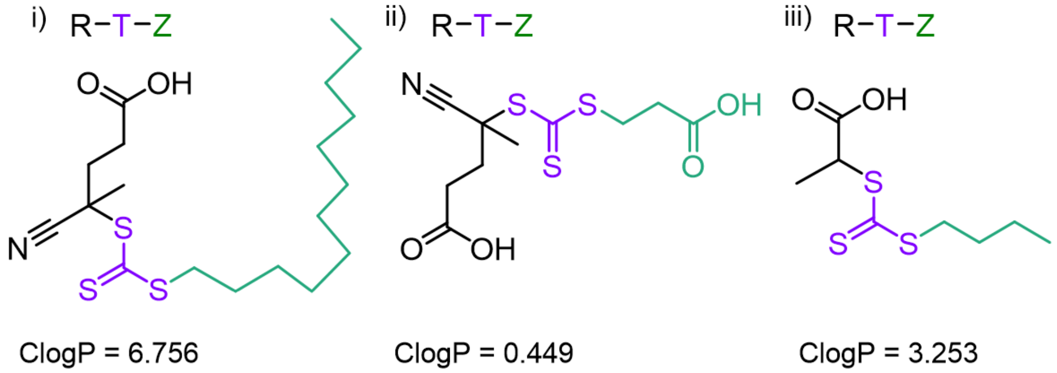

Figure S1. RAFT agents selected for SMA synthesis: i) Long hydrophobic RAFT agent 4-cyano-4-[(dodecylsulfanylthiocarbonyl)sulfanyl]pentanoic acid, ii) hydrophilic RAFT agent 4-((((2-carboxyethyl)thio)carbonothioyl)thio)-4-cyanopentanoic acid and iii) short hydrophobic RAFT agent 2-(butylthiocarbonythioythiol) propanoic acid. Trithiocarbonate groups are highlighted in purple and (Z)-end-groups are highlighted in green. Associated ClogP values, indicative of overall hydrophobicity, were estimated with ChemDraw v.22.2.0 software.

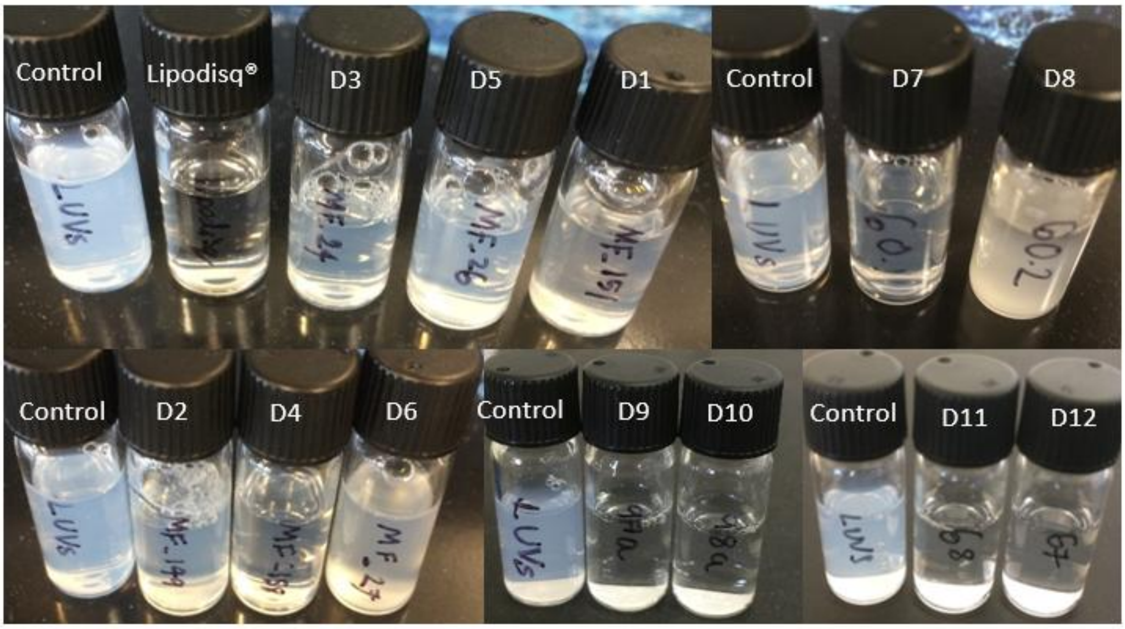

Figure S2. Turbidity observations for all samples containing 320 µL of either buffer (for the LUV negative control), 2.5 mM diblock SMA copolymer or ~3.5 mM Lipodisq® (positive control) added to 800 µL of 5mM DMPC LUVs (0.2:1 polymer:lipid ratio) and incubated at ~26 °C for 24 hours.

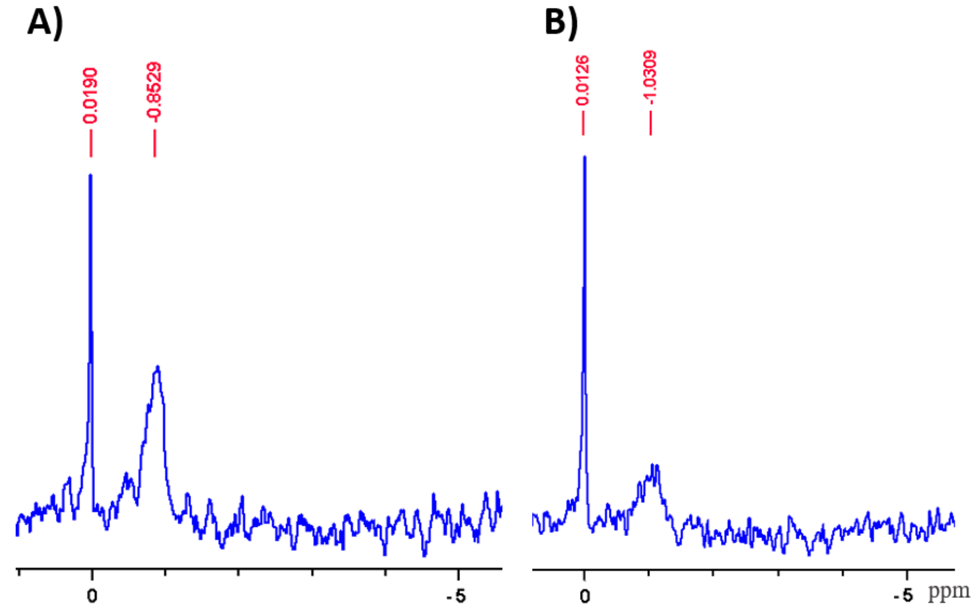

Figure S3. ^{31}P NMR data from equilibrated 0.2:1 SMA:DMPC (molar ratio) samples using **A) D10** (hydrophilic terminated) diblock SMA (M_n = 5,300) and **B) D12** (end-group removed) diblock SMA (M_n = 5,100).

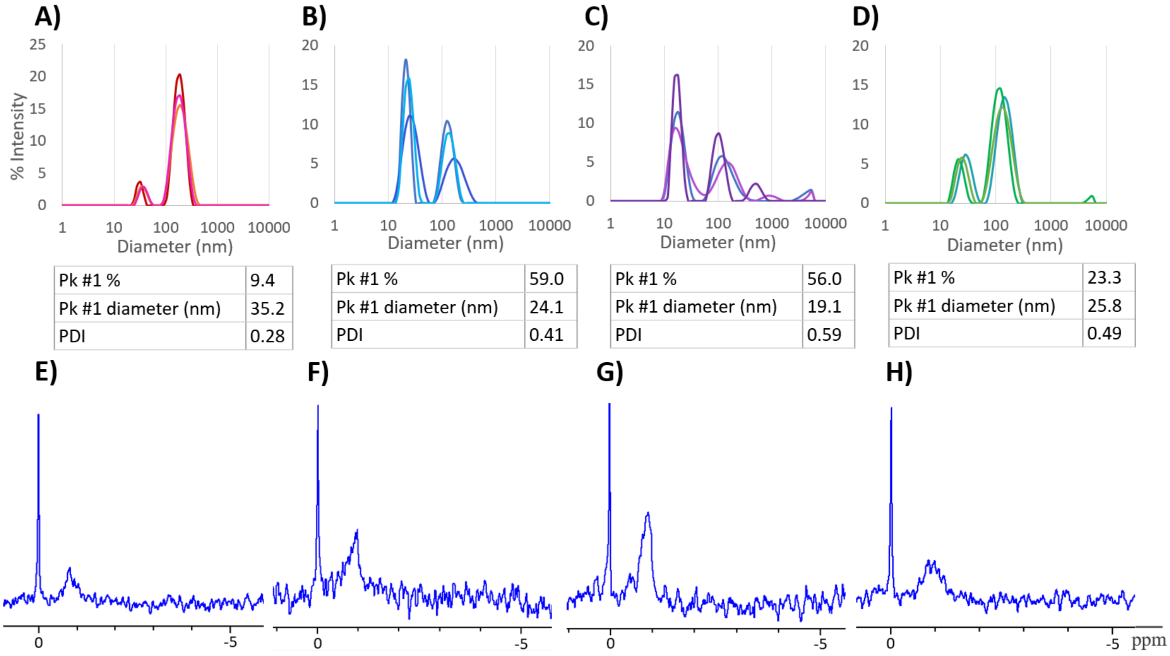

Figure S4. Intensity weighted DLS comparison **(A-D)** of SMALP diameter and formation efficiency for **A) D4**, **B) D9**, **C) D10**, and **D) D11** each added to DMPC LUVs in a 0.2:1 polymer:lipid molar ratio. Pk #1 % and Pk #1 diameter refer to the % scattering intensity and diameter of the smaller size population nanodisc peak, and PDI denotes the polydispersity index. Corresponding ^{31}P NMR spectra **(E-H)** are shown for **E) D4**, **F) D9**, **G) D10** and **H) D11** SMALPs each displaying a nanodisc peak at ~-1.0 ppm alongside an external H_3PO_4 reference peak at ~0 ppm.

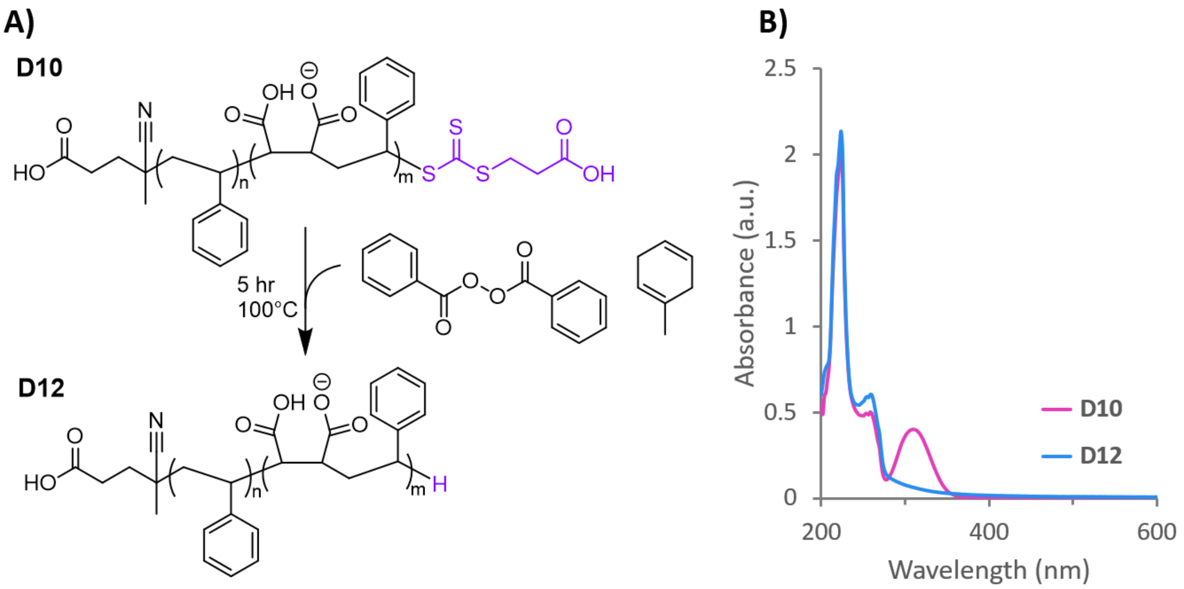

Figure S5. A) Radical-induced reduction reaction scheme used in polymer synthesis. **D10** SMA was converted to **D12**, the derivative without the trithiocarbonate and (Z)-end-group, by reaction with excess benzoyl peroxide radical initiator and hydrogen donor 1-methyl-1,4-cyclohexadiene in dioxane solvent. **B)** UV-visible absorbance spectra of hydrophilic diblock SMA (**D10**) in pink against end-group removed diblock SMA (**D12**) in 50 mM Tris-HCl buffer (pH = 8.00, 150 mM NaCl).

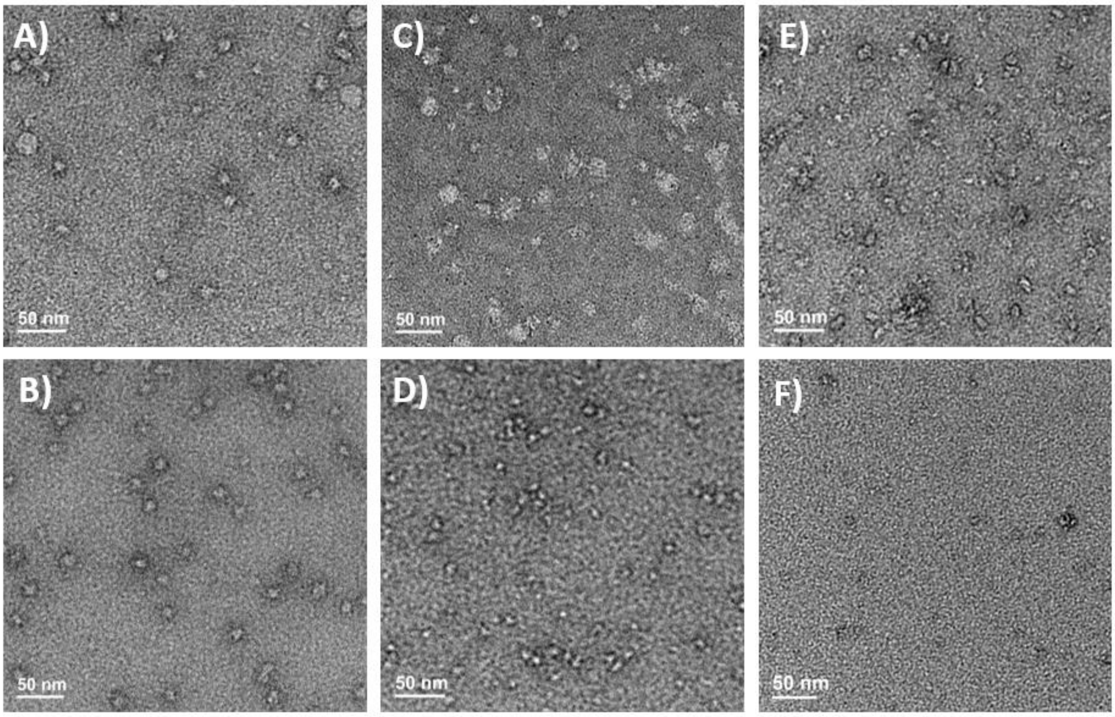

Figure S6. TEM images of **A) D3** SMA ((Z)-end-group = C_4H_9) incubated with DMPC LUVs at 0.2:1 polymer:lipid and **B) D3** SMA at the same polymer concentration (50 µM). **C) D11** SMA ((Z)-end-group = C_4H_9) incubated with DMPC LUVs at 0.2:1 polymer:lipid and **D) D11** SMA at the same polymer concentration (50 µM). **E) D10** SMA ((Z)-end-group = C_2H_4-COOH) incubated with DMPC LUVs at 0.2:1 polymer:lipid and **F) D10** SMA at the same polymer concentration (50 µM).

Table S1. Sequence design for diblock SMA copolymer library including target number average molecular weight (M_n), target styrene:maleic acid ratio (Sty:MA) as well as R, *a*-bock, *b*-block, T and Z chemical compositions within the R-*a*-*b*-T-Z diblock structure. Components of the diblock SMA sequence are colour coded according to whether they are hydrophobic (yellow), hydrophilic (blue), or a mixture of hydrophobic and hydrophilic (green).

Diblock SMA	Target M_n	Target Sty:MA	R	*a*-block	*b*-block	T	Z
**D1**	12,000	2.0:1.0	-C(CH$_3$)(CN)-CH$_2$-COOH	Sty-*alt*-MA	Sty	S-C(=S)-S	$C_{12}H_{25}$
**D2**	12,000	2.0:1.0	-C(CH$_3$)(CN)-CH$_2$-COOH	Sty	Sty-*alt*-MA	S-C(=S)-S	$C_{12}H_{25}$
**D3**	12,000	2.0:1.0	-C(CH$_3$)(H)-COOH	Sty	Sty-*alt*-MA	S-C(=S)-S	C_4H_9
**D4**	12,000	2.0:1.0	-C(CH$_3$)(CN)-CH$_2$-COOH	Sty	Sty-*alt*-MA	S-C(=S)-S	C_2H_4-COOH
**D5**	14,000	2.0:1.0	-C(CH$_3$)(CN)-CH$_2$-COOH	Sty	Sty-*alt*-MA	S-C(=S)-S	C_2H_4-COOH
**D6**	12,000	2.0:1.0	-C(CH$_3$)(CN)-CH$_2$-COOH	Sty	Sty-*alt*-MA	S-C(=S)-S	C_2H_4-COOH
**D7**	12,000	2.0:1.0	-C(CH$_3$)(CN)-CH$_2$-COOH	Sty	Sty-*alt*-MA	S-C(=S)-S	C_2H_4-COOH
**D8**	12,000	2.0:1.0	-C(CH$_3$)(CN)-CH$_2$-COOH	Sty	Sty-*alt*-MA	S-C(=S)-S	$C_{12}H_{25}$
**D9**	10,200	1.5:1.0	-C(CH$_3$)(CN)-CH$_2$-COOH	Sty	Sty-*alt*-MA	S-C(=S)-S	C_2H_4-COOH
**D10**	6,100	2.0:1.0	-C(CH$_3$)(CN)-CH$_2$-COOH	Sty	Sty-*alt*-MA	S-C(=S)-S	C_2H_4-COOH
**D11**	6,100	2.0:1.0	-C(CH$_3$)(H)-COOH	Sty	Sty-*alt*-MA	S-C(=S)-S	C_4H_9
**D12**	5,900	2.0:1.0	-C(CH$_3$)(CN)-CH$_2$-COOH	Sty	Sty-*alt*-MA	none	none

Supplementary Data: Gold Tethering Studies of SMALPs

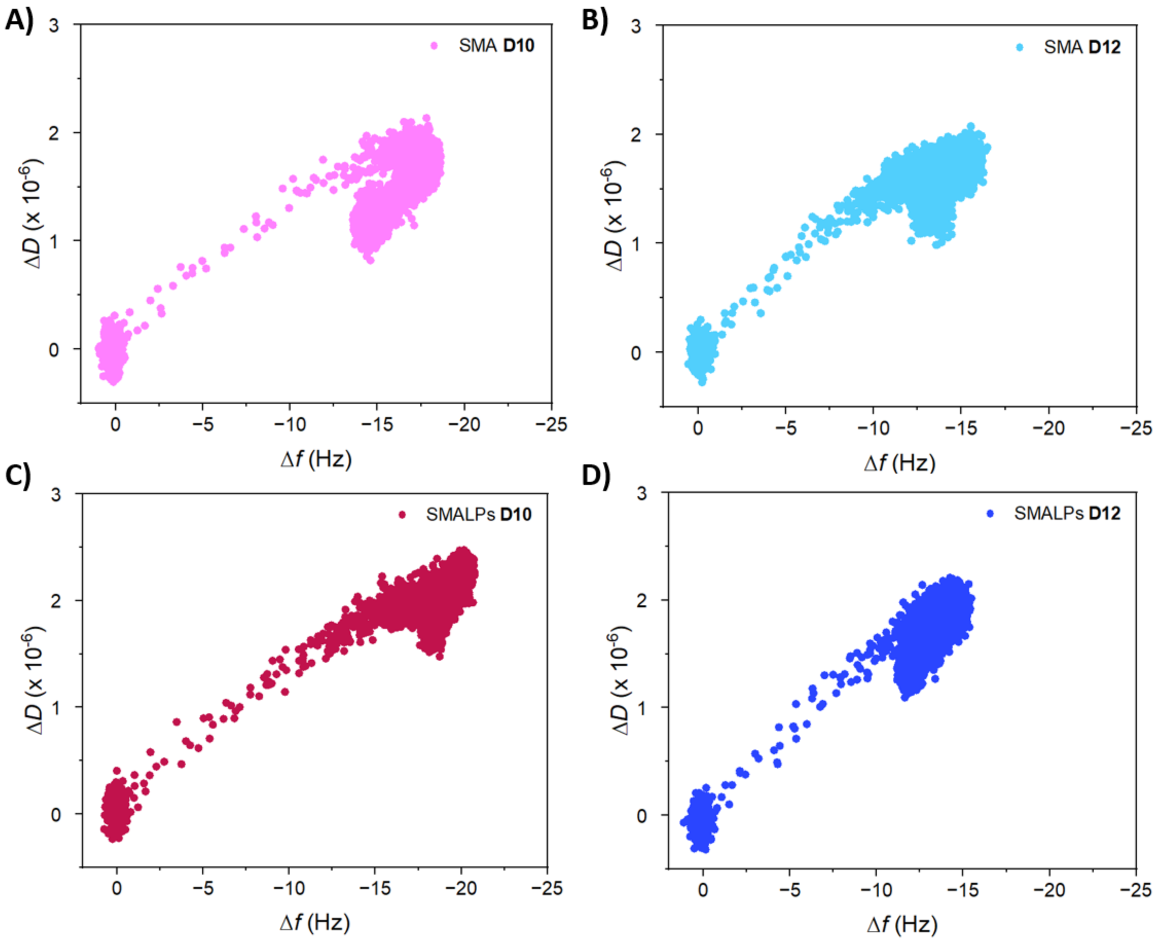

Figure S7. Plots of change in dissipation ($\Delta D7$(x 10^{-6})) versus change in frequency ($\Delta f7$(Hz)) for individual QCM-D experiments comparing **A)** 50 µM SMA **D10** (pink), **B)** 50 µM SMA **D12** (light blue), **C)** 0.2:1 (mol/mol) SMA:DMPC **D10** with 50 µM polymer (red) and **D)** 0.2:1 (mol/mol) SMA:DMPC **D12** with 50 µM polymer (dark blue).

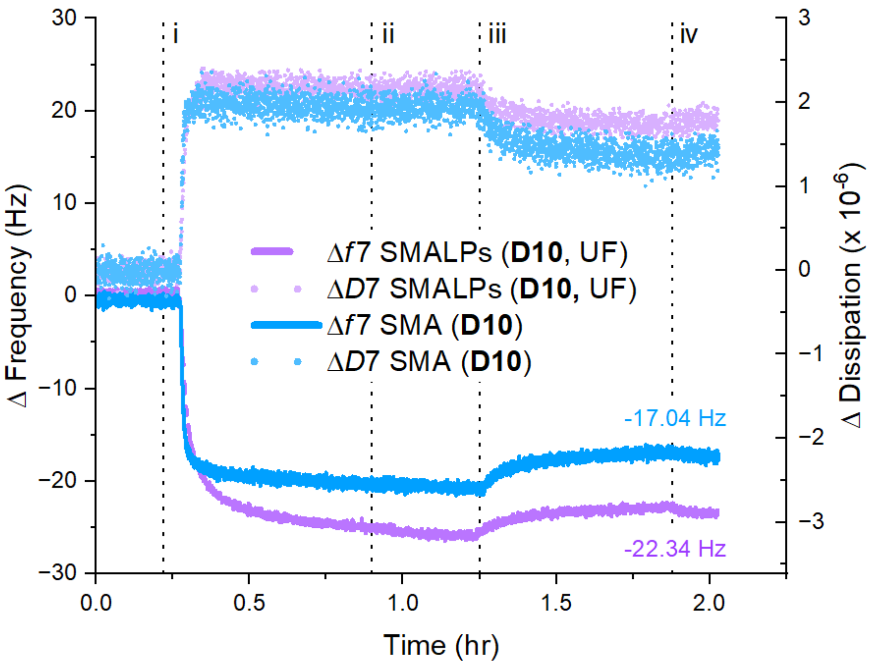

Figure S8. Comparison of QCM-D gold-binding experiments using 50 µM **D10** SMA (light blue) and **D10** SMALPs adjusted to 50 µM **D10** SMA content (purple) after ultracentrifugation (100,000 x *g*, 60 min, 4 °C) proceeded by 4 cycles of ultrafiltration with 10 kDa MWCO filters and a further 4 cycles of ultrafiltration with 30 kDa MWCO filters to purify SMALPs from SMA micelles. The SMALP solution was assembled with 0.2:1 (mol/mol) SMA:DMPC and incubated for 24 hr at 26 °C. Stages throughout the duration of the measurement are marked: i) SMA or SMALP addition, ii) no flow (static), iii) buffer wash and iv) no flow (static). The standard time-points for each stage are denoted by a black dotted vertical line. Time dependent Δ frequency (Hz) and Δ dissipation (x 10^{-6}) readings are shown for the 7th harmonic (Δ*f*7 and Δ*D*7). Δ*f*7 values for SMA and SMALPs at point iv) show an enhanced mass deposition evident for **D10** SMALPs.

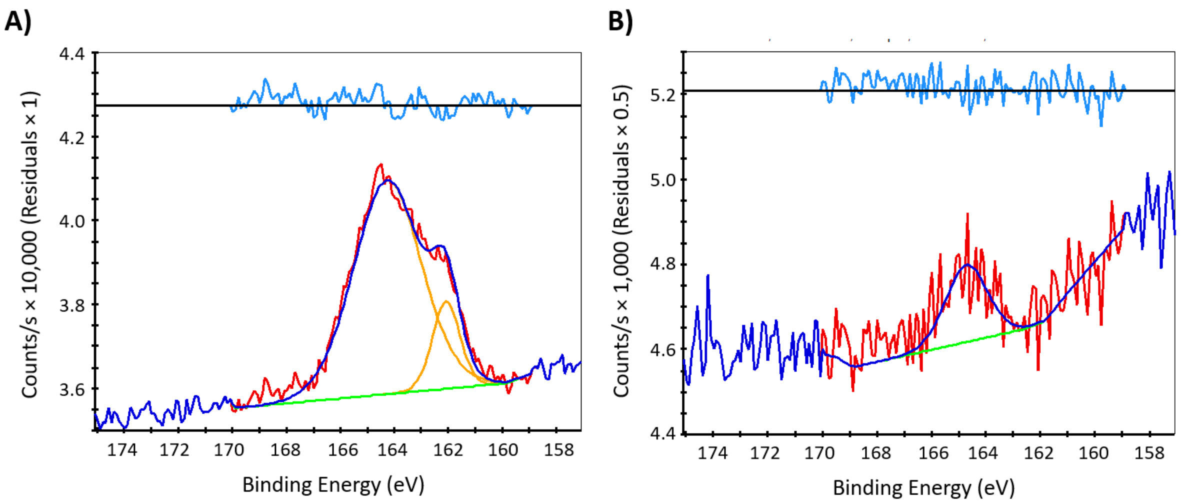

Figure S9. XPS sulfur scans (between 157-175 eV) against manually fitted curves and residuals derived using Avantage software for **A)** hydrophilic RAFT agent (HOOC-C_2H_4-RAFT) and **B)** 0.2:1 (mol/mol) SMA:DMPC SMALPs (**D10**) drop-casted onto gold-coated silicon wafers.

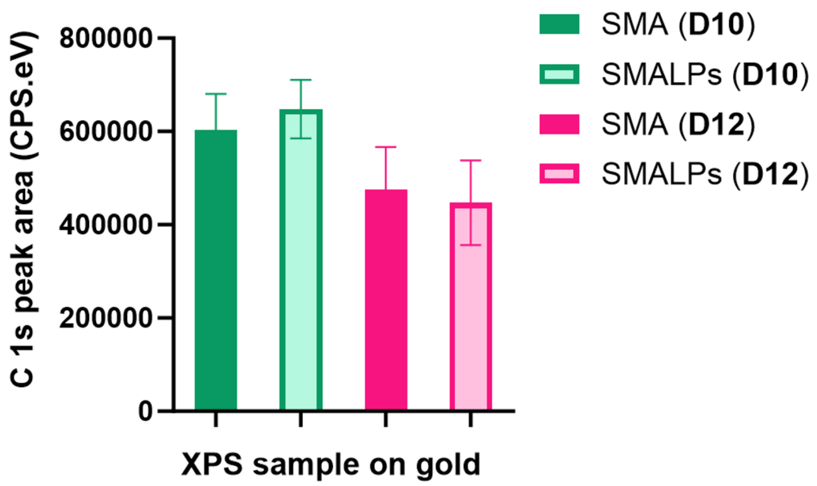

Figure S10. XPS measurement of carbon content bound to gold coated silicon wafers after treatment with diblock SMA copolymers or corresponding 0.2:1 (mol/mol) SMA:DMPC diblock SMALPs comprising **D10** (hydrophilic (Z)-end-group terminated) or **D12** (end-group removed) SMA (0.25 mM polymer). Samples were added onto gold-coated wafers before washing with buffer and ultrapure water. The mean carbon (C) 1s peak area is shown from triplicate survey scans after a baseline subtraction using the mean C 1s peak area of a buffer treated gold-coated wafer control.

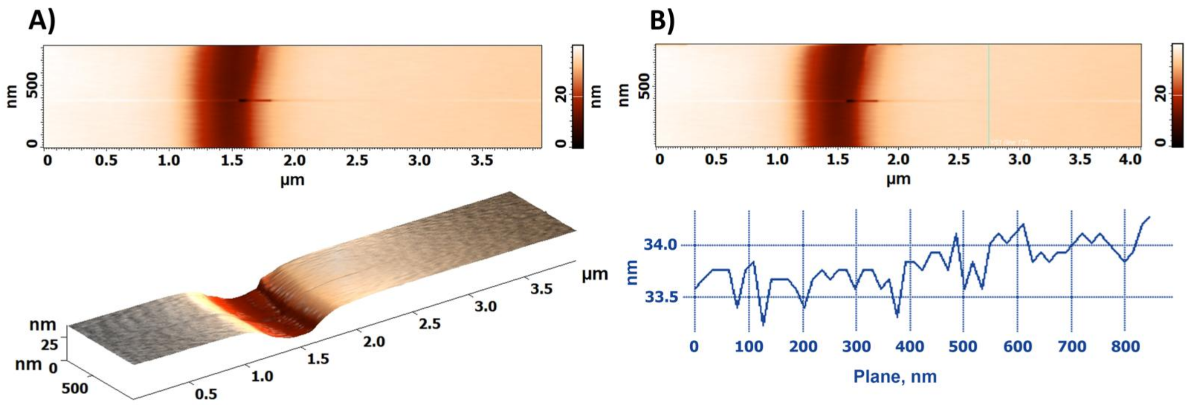

Figure S11. AFM images of a gold coated silicon wafer (control) including **A)** 2D z-height contrast topographical image (above), accompanying 3D representation (below). **B)** 2D z-height contrast topographical image with an intersecting plane (above) for which a cross section plot is given (below).

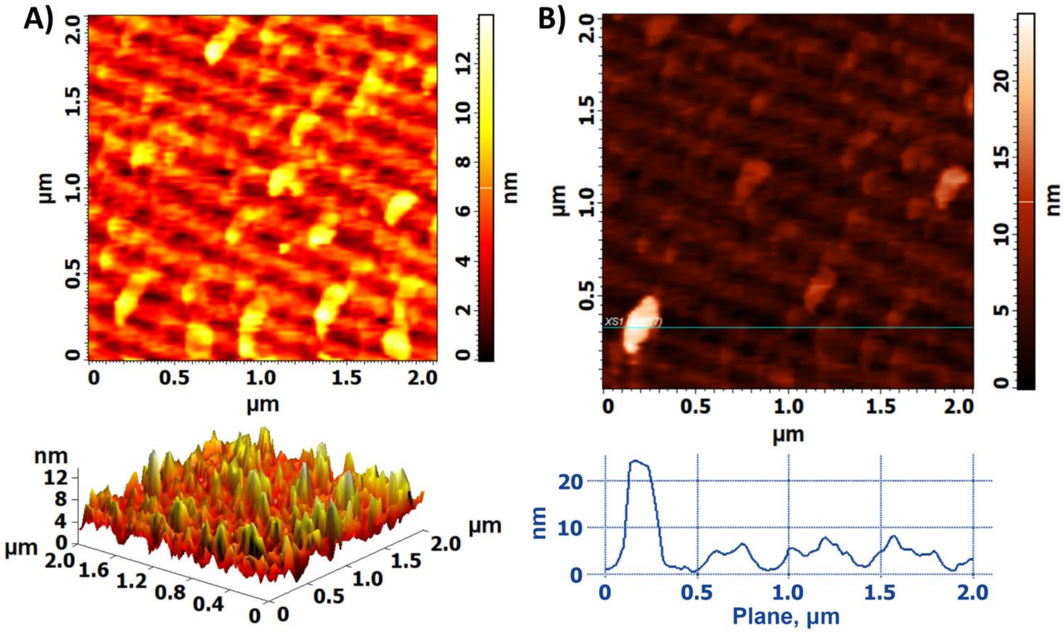

Figure S12. AFM images of diblock SMA (**D10**) blotted onto a gold coated silicon wafer including **A)** 2D z-height contrast topographical image and an accompanying 3D representation (below) and, **B)** 2D z-height contrast topographical image with an intersecting plane for which a cross-section plot is given (below).

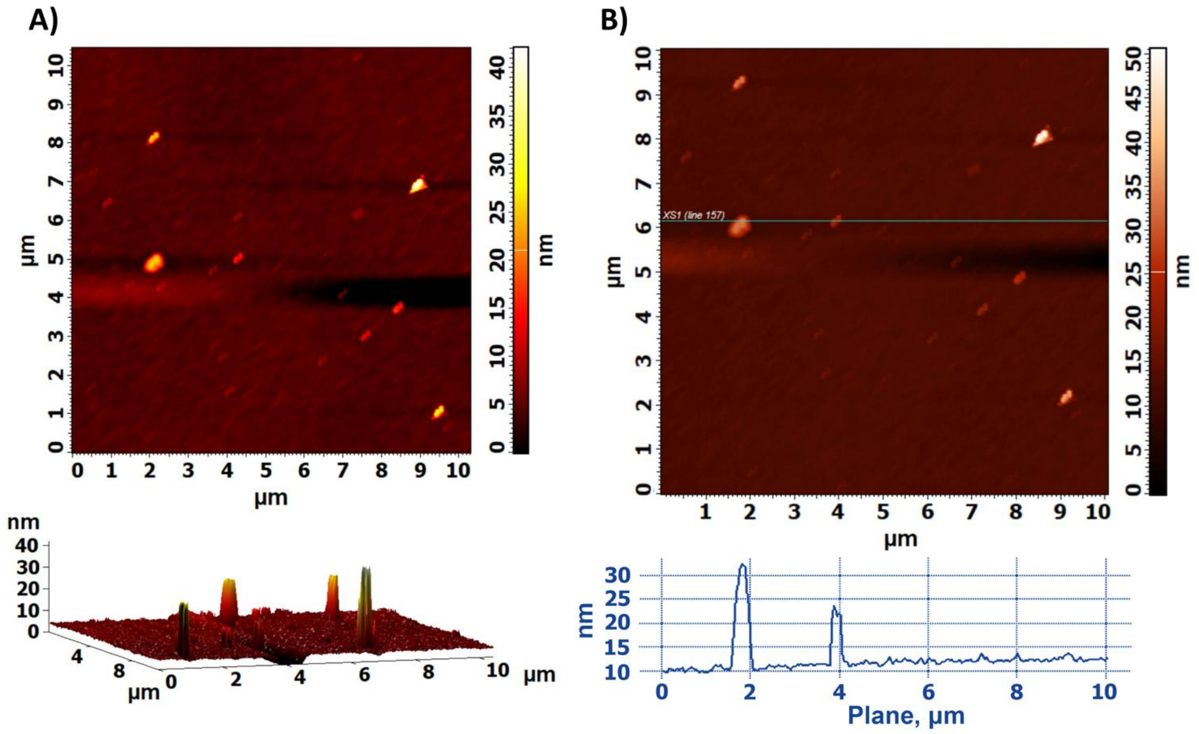

Figure S13. AFM images of diblock SMA (**D12**) blotted onto a gold coated silicon wafer including **A)** 2D z-height contrast topographical image and 3D representation (below) and, **B)** 2D z-height contrast topographical image with an intersecting plane for which a cross section plot is given (below).

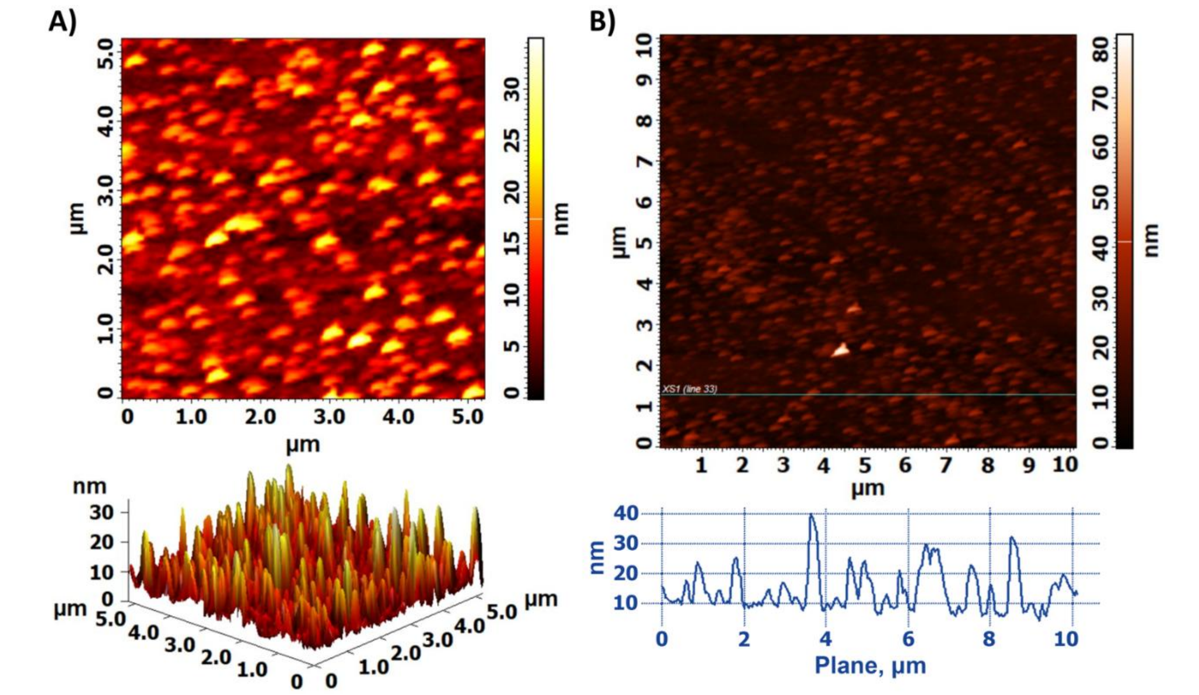

Figure S14. AFM images of 0.2:1 (mol/mol) SMA:DMPC SMALPs (**D12**) added onto a gold-coated silicon wafer and rigorously washed with buffer and ultrapure water. **A)** 2D z-height contrast topographical image and accompanying 3D representation (below) and, **B)** 2D z-height contrast topographical image with an intersecting plane for which a cross section plot is given (below).

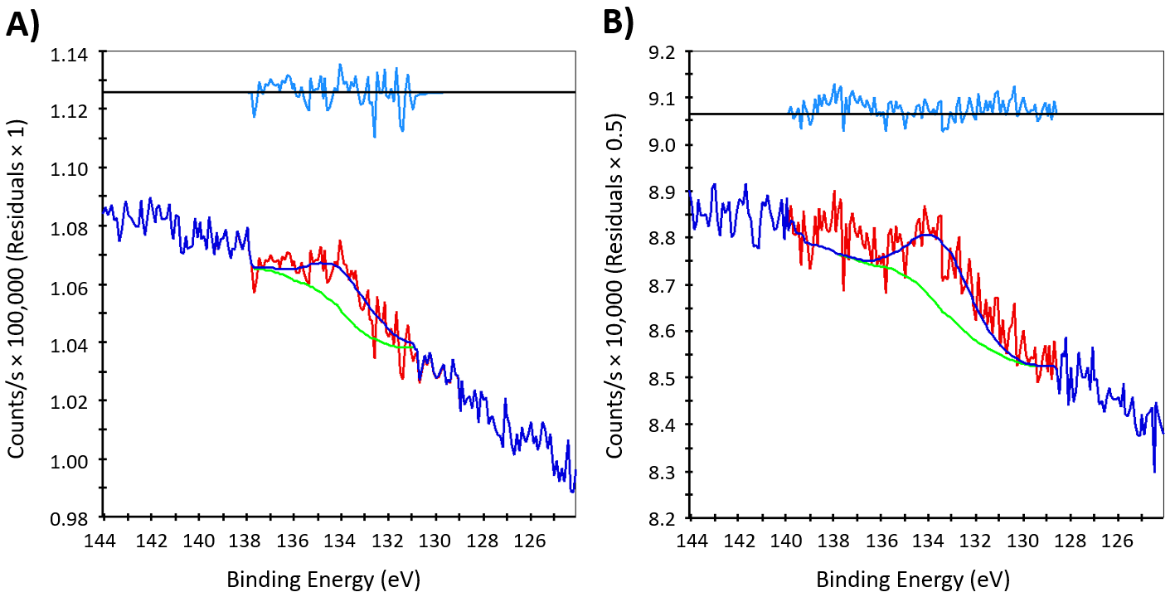

Figure S15. XPS phosphorus scans (between 124-144 eV) against manually fitted curves and residuals derived using Avantage software for **A)** 0.2:1 (mol/mol) SMA:DMPC SMALPs (**D10**) and **B)** 0.2:1 (mol/mol) SMA:DMPC SMALPs (**D12**) solutions deposited onto gold coated silicon wafers and washed with HS PBS buffer and then with water.
